# Tumor-infiltrating B cells evolve towards interferon-rich trajectories aligned with immunotherapy in melanoma

**DOI:** 10.1186/s13046-026-03797-1

**Published:** 2026-08-07

**Authors:** Katie Stoker, Roman Laddach, Lucy Booth, Silvia Crescioli, Oscar Maiques, Rebecca Adams, Jitesh Chauhan, Alexander Stewart, Dinethri Patel, Amanda Fitzpatrick, Yin Wu, Jenny L. C. Geh, Omar Salem, Alastair D MacKenzie Ross, Hawys Lloyd-Hughes, Sean Whittaker, Joseph Ng, Franca Fraternali, David Kipling, Deborah K. Dunn-Walters, Katie E. Lacy, Thomas J. Tull, Sophia Tsoka, Sophia N. Karagiannis

**Affiliations:** 1 St. John’s Institute of Dermatology, School of Basic and Medical Biosciences &, KHP Centre for Translational Medicine, Guy’s Hospital, King’s College London, 9th Floor, Guy’s Hospital, Tower Wing, London, SE1 9RT UK; 2 Department of Informatics, Faculty of Natural, Mathematical and Engineering Sciences, King’s College London, Bush House, Strand Campus, London, WC2B 4BG UK; 3 Barts Cancer Institute, Queen Mary University of London, John Vane Science Building, Charterhouse Square, London, EC1M 6BQ UK; 4 School of Biosciences and Medicine, University of Surrey, Guildford, GU2 7XH UK; 5 Department of Medical Oncology, Guy’s and St Thomas’ Hospitals, London, SE1 9RT UK; 6 Breast Cancer Now Research Unit, School of Cancer and Pharmaceutical Sciences, King’s College London, Innovation Hub, Guy’s Cancer Centre, SE1 9RT London, UK; 7 Centre for Inflammation Biology and Cancer Immunology, School of Immunology & Microbial Sciences, King’s College London, London, SE1 9RT UK; 8 St John’s Institute of Dermatology, Guy’s, and St. Thomas’ Hospitals NHS Foundation Trust, London, SE1 9RT UK; 9 Department of Plastic Surgery, Guy’s and St. Thomas’ Hospitals NHS Foundation Trust, London, SE1 7EH UK; 10 Randall Centre for Cell and Molecular Biophysics, School of Basic and Medical Biosciences, King’s College London, London, SE1 9RT UK; 11 Department of Structural and Molecular Biology, Division of Biosciences and Institute of Structural and Molecular Biology, University College London, London, WC1E 6BT UK; 12 Faculty of Health and Medical Sciences, University of Surrey, Guildford, Surrey GU2 7XH UK

**Keywords:** Melanoma, Cancer immunotherapy, B Cells, Antibodies, Singe cell RNA-Seq, Bulk RNA-Seq, Spatial Transcriptomics, Checkpoint inhibitors

## Abstract

**Background:**

B cells are increasingly recognized regulators of antitumor immunity in melanoma, yet their differentiation states, lineage dynamics, spatial organization and integration within the immune microenvironment, and how these are influenced with immunotherapy remain incompletely defined.

**Methods:**

We conducted deep profiling of tumor-resident B cell differentiation and evolutionary features in the melanoma tumor microenvironment using bulk, single-cell and spatial transcriptomic analyses, long-read antibody sequencing and mass cytometry (Cytometry by Time-Of-Flight, CyTOF). We characterized B cell differentiation trajectories, class-switching patterns, antibody isotype usage, signaling pathways, spatial niches against clinicopathological features and immune cell communication, before and after checkpoint blockade and in clinically defined responders and non-responders.

**Results:**

Melanoma-infiltrating B cells were enriched across primary and metastatic lesions, were preferentially associated with immune-rich tumors and correlated with T and natural killer (NK) cell densities. Long-read immunoglobulin sequencing demonstrated recurrent class switch recombination (CSR) trajectories with predominant IgG and IgA isotype usage, confirmed by bulk transcriptomic analyses. Across independent single-cell and bulk transcriptomic datasets we identified conserved class-switching, B cell receptor (BCR) activation, and pseudotime analyses showed differentiation trajectories progressing from naïve and memory states toward interferon (IFN)-driven terminal differentiation trajectories that persisted before and after immunotherapy. CyTOF analyses independently confirmed proliferative, germinal center (GC)-like, memory and class-switched B cell subsets, suggesting conserved inflammatory differentiation endpoints. Spatial transcriptomic deconvolution identified IFN-rich B cells within tertiary lymphoid structures (TLS). Following immunotherapy, B cells retained class-switched trajectories, displayed enriched GC-associated profiles, enhanced crosstalk with T cells and Dendritic Cells (DCs) and preserved or induced IFN-rich IgM + /IgD + or IgG4 + phenotypes, suggesting persistence or emergence of non-class switched and atypical class-switched isotype profiles. Transcriptomic analyses of B cells pointed to upregulated antigen-driven stimulation and BCR signaling, whereas in non-responders displayed IFN-rich B cells, IFN-associated genes, and IFN-pathway enrichment.

**Conclusions:**

Melanoma-infiltrating B cells follow class-switched differentiation trajectories culminating in IFN signaling, atypical antibody expressing states that persist or emerge after immunotherapy, aligning dynamic B cell evolution and atypical lineage traits with immunotherapy.

**Supplementary Information:**

The online version contains supplementary material available at 10.1186/s13046-026-03797-1.

## Introduction

B cells are increasingly recognized as key regulators of antitumor immunity in melanoma, influencing T cell activation, tertiary lymphoid structure (TLS) formation, and responses to immune checkpoint inhibitor immunotherapy. Yet differentiation, lineage dynamics and spatial organization of tumor resident B cells, and how these integrate with the immune microenvironment to shape therapeutic outcomes, remain poorly defined. Clarifying these processes is essential for elucidating treatment response and resistance.

Local microenvironmental cues in melanoma may shape B cell differentiation, subset frequency and migration. B cells may undergo class switching and expansion to form distinct clonal families [11, 15] and their expressed antibodies may be associated with variable clinical outcomes [6]. The formation of immune cell aggregates and TLS may indicate organized B cell responses driven through T cell and dendritic cell (DC) interactions. On the other hand, atypical, alternatively-activated B cell responses [43] may denote less potent responses driven by inflammatory signals.

We previously reported clonal expansion, class switch recombination (CSR), and somatic hypermutation (SHM) driven memory B cell responses in melanoma. Simultaneously, we found evidence of receptor revision and expression of low-affinity antibodies to several self-antigens. These phenomena are often described in B cells associated with autoimmunity [12, 32, 44]. B cell clonal families showed a bias towards alternatively-activated IgG4 and IgA immunoglobulin isotypes, in line with B cell responses observed during chronic inflammatory or autoimmune conditions [34, 41], while increased patient survival probability was associated with higher intra-tumoral IgG expression [12]. Combined, these features denote activated on one hand, but diverted autoimmune-like and humoral immune responses on the other.

In this study, we undertake deep profiling of tumor-resident B cell differentiation and evolutionary features in melanoma and examine how these are altered by immunotherapy or with treatment response. We analyze B cell evolution and interaction of B cells with other immune cells in the melanoma tumor microenvironment (TME), especially T cells and DCs required for Germinal Centre (GC) and TLS formation. We further interrogate B cell subset spatial distribution and assembly. By bulk-RNA sequencing analysis, we characterize the humoral compartment in normal skin, melanoma primary lesions, and cutaneous or visceral metastasis sites. By single-cell (sc)RNA-seq and spatial transcriptomic analyses, we study B cell differentiation states, evolution trajectories, and B cell subset crosstalk and localization with other immune cells required for GC formation within tumors. By scRNA-seq analysis of tumors at baseline and following immunotherapy, we interrogate tumor-associated immune lineage traits from responders and non-responders to checkpoint inhibitor immunotherapy. Our study provides a spatial, evolutionary and cellular crosstalk assessment of B cells within the melanoma TME and B cell lineage dynamics in immunotherapy.

## Materials and methods

### Bulk RNA-sequencing data

An integrated bulk RNA-seq dataset TCGA- GTEx containing data from The Cancer Genome Atlas (TCGA, https://www.cancer.gov/tcga) (*n* = 451) and non-diseased tissues (Genotype-Tissue Expression, GTEx) (*n* = 556) from UCSF XENA (xenabrowser.net). The FPKM-normalized (fragments per kilobase of transcript per million mapped reads) dataset was downloaded from xenabrowser.net (Goldman et al., 2020). Healthy skin data used for the analyses described in this manuscript were obtained from the Genotyping-Tissue Expression (GTEx) Portal (https://www.gtexportal.org/) (*n* = 556). The ensemble gene IDs were converted to gene names using the biomaRt (RRID:SCR_019214) package (Durinck et al., 2009). In the case of multiple IDs mapped to a single gene, TMP values were averaged. Patients were split based on tumor annotation into primary (*n* = 91) and metastatic (*n* = 360). The metastatic group was further divided based on the site of resection/biopsy into metastatic skin (*n* = 116), visceral (*n* = 36) and lymph node (*n* = 208).

To study the immune composition of melanoma, the TPM-normalized dataset was used as an input for the consensusTMEAnalysis [21], and the normalized enrichment score was used for downstream analyses. We applied the ConsesusTME tool [21] to deconvolute bulk transcriptomic data, since this tool provides information about enrichment of 17 different cell populations, including 15 immune populations. The enrichment was normalized, allowing for comparison of the same cell population across all samples.

### Processing of single-cell RNA-sequencing data

The scRNA-seq melanoma dataset from Li et. al., was downloaded from Gene Expression Omnibus (GEO) (RRID:SCR_005012) under the accession code GSE123139 [28]. Additional information about the tissue was obtained from TISCH repository (tisch.comp-genomics.org). The scRNA-seq melanoma dataset from Sade-Feldman et al., was downloaded from GEO under the accession code GSE120575 [33]. The data from GSE123139 and GSE120575 were split into pre- and post-treatment samples and analyzed separately. Pre-processing and clustering were performed using the Seurat (v5.1.0) pipeline [18]. Seurat objects were log normalized using NormalizeData with a scale factor of 10,000, data were scaled and centred. Variable genes (2000) were identified (function FindVariableFeatures, method = “vst”) and were used for principal component analysis (PCA) using elbow plots to select the top principal components for clustering using the FindNeighbors and FindClusters functions. Clusters identified as B cells and plasma cells were used for subsequent analyses. For clustering of B cells and plasma cells, heavy and light antibody chain genes were removed from variable features to minimize the formation of clonotypic clusters driven by specific heavy or light chain expression.

### Differential gene expression, gene over-representation and trajectory analysis

Analyses of differentially expressed genes (DEG) using FindMarkers were used to calculate the top DEGs per B cell subset, DEGs were filtered for padj ≤ 0.05. Significant DEGs for the B cell subsets were analyzed for functional enrichment using gprofiler2 (v0.2.3) [26]. Trajectories between B cell populations were identified using the package Slingshot (v2.12) [37] in an unsupervised manner without specifying the starting cluster. The package liana (v0.1.14) [13] was used to assess the potential cell–cell interaction axes.

### Human melanoma sample collection for spatial transcriptomics analyses

Spatial transcriptomic analyses in human melanoma specimens from nine patients were collected with informed written consent in accordance with the Declaration of Helsinki. Both male and female patients were selected. Age of patients ranged from 45 to 79 years. Types of tumors include primary melanoma, in-transit metastasis, and locoregional lymph node metastasis. Tumors were selected irrespective of biopsy location or stage of disease. The study was conducted at King’s College London, Guy’s and St Thomas’ NHS Foundation Trust (REC reference: 08/H0804/139 approved by London Bridge NRES committee; REC reference: 16/LO/0366 (MISST, melanoma) approved by London-Central NRES Committee). OCT frozen samples were processed using the 10X Genomics Visium platform, according to manufacturer’s instructions. The 10X Genomics Space Ranger (v2.1.1) count pipeline using the human reference transcriptome (refdata-gex-GRCh38-2020-A) was used to process the Visium data. The Load10XSpatial was used to load the Space Ranger output for each sample as a Seurat object. Normalization was performed using SCTransform [16].

Single-cell RNA-seq data was used to deconvolute the spatial transcriptomic (*n* = 8) samples using robust cell type decomposition (RCTD) in full-mode, where each transcriptomic spot is 55 µm and contains up to 10 cells [9]. One tumor sample was not suitable for deconvolution due to tissue size and folding. RCTD was selected for spatial deconvolution because it explicitly models and corrects for platform effects between the scRNA-seq reference and spatial transcriptomics data.

To maximize biological relevance and minimize confounding variability, we used a scRNA-seq reference derived from the same sample type (melanoma), as tissue- and disease-matched references are known to improve deconvolution accuracy relative to references from unrelated tissue contexts. Seurat’s AddModuleScore function was used to calculate enrichment scores of a tonsil derived single-cell RNA sequencing GC signature from Hamish W. King et al. [25], and gene sets from the Molecular Signatures Database (MsigDB) [31, 39].

### Histological annotation of spatial transcriptomic samples

Histological annotations performed at a single-cell resolution were conducted using QuPath [3, 29]. Distribution of immune, stroma and tumor cells, and annotation of specific tumor regions including the tumor body, tumor invasive front and TLS, were conducted by a trained dermatopathologist. Histological annotation was mapped to the Visium transcriptomic spots [29].

### Long-read sequencing, antibody isotype distribution and class-switch recombination in spatial transcriptomic samples

Long-read antibody repertoire sequences from the tumor samples used for spatial transcriptomics were generated and full-length antibody sequences were annotated using IMGT/HighV-Quest [27], as before. To analyze antibody distribution in each of the spatial transcriptomic samples, transcriptomic data from each of the melanoma samples was combined and the percentage of antibody isotypes were calculated using the isotype.R script (https://github.com/josef0731/melanoma-ig/blob/main/bcr-repertoire/rep-featues/isotype.R). Class-switch recombination events were analyzed using the BrepPhylo package (https://github.com/Fraternalilab/BrepPhylo) [36].

**Fig. 1:**
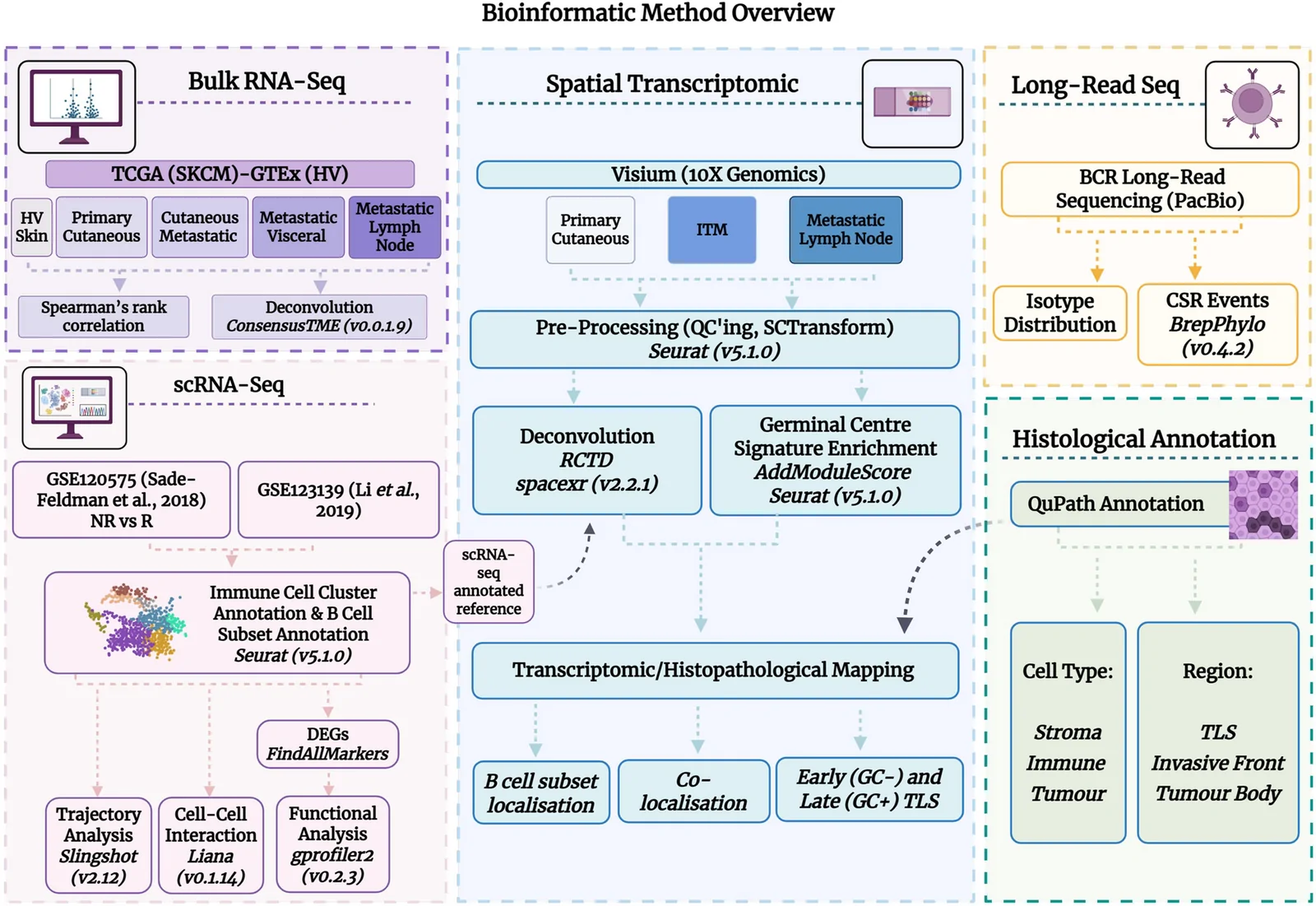
Bioinformatics pipeline for the analysis of bulk, single-cell (scRNA-seq) and spatial RNA transcriptomics data, long-read immunoglobulin, and histological data from melanoma samples. Bulk RNA-seq was analysed to determine cell type correlation and enrichment in melanoma samples. Single-cell RNA-seq data was analyzed to dissect the B cell profiles of pre- and post-treatment melanoma tumors. B cell subset signatures were validated using differential gene expression analysis, functional enrichment analysis, and enrichment analysis in specific B cell clusters using known transcriptomic signatures. The annotated scRNA-seq reference was used to deconvolute spatial transcriptomic melanoma samples to explore the localisation of immune cell types across the tumor, and within various histological regions annotated in QuPath. Long-read sequencing was used to analyse antibody isotype distribution and CSR events. Healthy volunteer (HV) skin, non-responder (NR), responder (R), germinal centre (GC), tertiary lymphoid structure (TLS), class-switch recombination (CSR), B cell receptor (BCR), in transit metastasis (ITM)

The bioinformatics pipelines and methods used in this study are summarized (Fig. 1). R (v4.1.1) was used to analyze pre-processed data. Datasets analyzed are outlined in Supplementary Table 1.

### Suspension mass cytometry of melanoma tumor-derived B cells

Human tissue samples were collected from tumors of four patients with melanoma (Supplementary Table 4). Tissues were minced with a scalpel followed by mechanical dissociation with gentle MACS dissociator in RPMI 1640 medium supplemented with 1 mM EDTA. The cell suspension was passed through a 100um cell strainer and cryopreserved as previously described [12, 43]. CyTOF was conducted for high-dimensional immunophenotyping analysis of B cells using a panel of antigen markers (Supplementary Table 5). Cell staining, data acquisition and FlowSOM analysis are detailed in supplementary materials and methods (Supplementary Materials and Methods 1.1, 1.2). FlowJo (RRID:SCR_008520) was used to manually gate live, singlet, intact cells that were CD19 + and CD27 +.

### Thawing of melanoma tumors for mass cytometry analyses

Cryovials of frozen single cell-suspended tumors were thawed in a 37 °C water bath. When only a few ice crystals remained, cells were aspirated with a sterile Pasteur pipette and transferred into 10 ml warmed RPMI 1640 + 20% FBS. Cells were washed with 50 ml RPMI 1640 and centrifuged at 300 g for 10 min. Cells were then incubated for 1 h at 37 °C, followed by another RPMI wash as above. PBMC counts were performed on a haemocytometer using trypan blue (1:10 in PBS).

### Mass cytometry cell staining and data acquisition

Thawed cells were placed in filter cap FACS tubes and washed with 3 ml MaxPar Cell Staining Buffer (Standard Biotools) at 800 g for 5 min. Pellets were resuspended in 80 µl buffer, transferred to FACS tubes, and incubated with 5 µl Fc Blocking Solution (Human TruStain FcX, BioLegend) for 10 min at room temperature. All antibodies were vortexed and centrifuged at 10,000 g for 5 min to remove aggregates before use. Surface antibodies were added, and samples were gently vortexed and incubated for 30 min at room temperature. Cells were washed with 1 ml MaxPar Cell Staining buffer, and 1uM Rhodium intercalator was used as a viability stain, added to each sample for 15 min at RT. Cells were washed as before, and fixed using 1 ml MaxPar Fix I buffer (Standard Biotools) at room temperature for 15 min, then washed twice in MaxPar Perm S permeabilisation buffer (Standard Biotools). The intracellular antibody cocktail was added and incubated 30 min at room temperature. After a wash in MaxPar buffer, cells were incubated overnight at 4 °C in intercalation solution containing DNA intercalator 103 Ir (Standard Biotools) in MaxPar Fix and Perm Buffer. Immediately prior to acquisition, cells were washed in MaxPar Cell Staining Buffer and washed twice in Milli Q water at 800 g for 5 min. Samples were resuspended in Milli Q water with polystyrene bead standards at 0.5 × 10⁶ cells/ml and run on a Helios™ Mass Cytometer.

### Mass cytometry and cluster analysis using the FlowSOM clustering algorithm

Raw data were received as.fcs files from the Helios™ Mass Cytometer. Files were normalized and concatenated using Fluidigm CyTOF Software and then uploaded to FlowJo for gating. EQ beads, DNA intercalator, and event length were used to discriminate intact DNA positive singlets from debris and aggregates. Rhodium live/dead staining identified live intact singlets. B cells were gated as CD45 + CD19 +, then refined to exclude doublets using the markers CD45, CD19, CD8a, CD4, CD3, CD16a to define a ‘True’ CD19 + population followed by CD27 +. After isolating the memory B cell CD19 + CD27 + population in FlowJo, the.fcs files were exported for downstream analysis in R. We used a modified script based on the CATALYST, diffCYT, FlowSOM, edgeR (RRID:SCR_012802), and flowCORE R packages. CD27 + B cell phenotypes were defined with an unsupervised FlowSOM clustering algorithm, which uses a self-organizing map to evaluate marker expression in high dimensional space and generate well segregated B cell subsets. Samples pooled for clustering yielded distinct metaclusters, which were annotated according to median scaled expression as previously described (Nowicka et al., 2019).

### Pooled bulk RNA sequencing

Single-cell-suspended melanoma tissue samples were thawed and stained for CyTOF acquisition as previously described [43], using metal-tagged antibodies (Supplementary Table 5). Cell acquisition was carried out using a Helios mass cytometer. Following acquisition.fcs files were normalised, concatenated and CD19 + B cells were gated using the FlowJo software: Iridium DNA intercalator determined intact DNA + singlets from debris and cell aggregates, Rhodium live/dead stain identified live intact singlets, CD19 + B cell populations were defined by CD19 + CD45 + cells, and then further refined to identify the true CD19 + population to exclude other cell types by using the markers: CD3 and CD16. CD27 was used to define CD27 ± B cells. CD19 + B cells and CD45 + lymphocytes were sorted from seven single cell-suspended melanoma tumor tissue samples using a FACS Aria cell sorter. RNA was extracted from each sample using the Direct-zol™ RNA Microprep kit (Zymo Research). Library construction, sequencing and data pre-processing were performed by Genewiz®, where sorted B cell RNA and sorted CD45 + RNA was pooled from donors. PolyA selection for mRNA was performed to remove ribosomal RNA. PolyA selection for mRNA was performed to remove ribosomal RNA. Libraries were sequenced on Illumina (approx. 20 million reads per sample).

Gene-level read counts were generated from the aligned BAM file using featureCounts, with exon features summarized to gene-level meta-features using the gene_id attribute from a GTF annotation. Ensembl/GENCODE gene identifiers were mapped to gene symbols using gene-level records extracted from the same GTF file used for quantification. Counts were normalized as counts per million (CPM) using edgeR package (v4.2.2) and the cpm function, followed by log2(CPM + 1) transformation, to correct for sequencing depth. Genes with fewer than 10 raw reads were flagged as below a reliable detection threshold.

A curated panel of type I (IFN-α, IFN-β, IFN-ε, IFN-κ, IFN-ω), type II (IFN-γ), and type III (IFN-λ) interferon ligands, their cognate receptors, canonical JAK-STAT signaling components (STAT1/2, IRF1/3/7/9), and downstream interferon-stimulated genes (ISG15, MX1/2, OAS1-3/OASL, IFIT1-3, IFI6/27/44/44L, IFITM1/3) was cross-referenced against normalized expression values to characterize interferon pathway activity in the pooled sample.

Single-sample gene set enrichment analysis (ssGSEA), implemented via the GSVA package (v1.52.3) (ssgseaParam/gsva), was used to derive pathway-level interferon activity scores from log2(CPM + 1)-normalized expression, using the Hallmark Interferon Alpha Response and Hallmark Interferon Gamma Response gene sets (MSigDB, retrieved via msigdbr, Homo sapiens). As this reflects a single pooled sample, the resulting ssGSEA score represents a single absolute enrichment value rather than a rank within a cohort. Bar plots of log2(CPM + 1) expression for interferon panel genes passing the detection threshold (raw count ≥ 10) were generated using ggplot2 (v4.0.3).

## Results

### Bioinformatics pipeline development to study B cells by bulk, single-cell and spatial transcriptomics analyses in histologically annotated melanoma samples

To interrogate B cell infiltration, localization, lymphoid assembly, evolutional trajectories and their associations with immunotherapy responses, we developed a computational pipeline (Fig. 1) which: (i) analyses integrated bulk RNA-seq (TCGA-SKCM) with normal skin (TARGET-GTEx) datasets and applied the ConsesusTME [21] tool to deconvolute RNA-seq data, with enrichment normalized to allow comparison of B cell populations across samples, alongside long-read immunoglobulin sequencing data to offer insight into antibody isotype distribution and CSR. (ii) Analyses the B cell profiles of two distinct scRNA-seq datasets to dissect cellular evolution pre- and post-treatment and assess B cell lineage traits [37] with immunotherapy outcomes [18]. (iii) Utilizes the annotated scRNA-seq reference to deconvolute [9] spatial transcriptomic melanoma samples. We additionally plotted the expression of pan-B cell markers using spatial feature plots and performed GC gene signature enrichment scoring directly on the spatial transcriptomic data, which provides a reference-independent, transcriptional readout of GC B cell activity. Spatial samples were used to explore the localization of immune cell types across the tumor, and within various histological regions, by histological annotation; and quantify B cell subset localization in tumor lesions by spatial transcriptomic and QuPath analyses [29].

### B cell and class-switch recombination markers are expressed across melanoma sites and IgG2 +, IgA1 +, IgG4 + and, IgG1 + B cells are the most observed

We assessed global B cell infiltration in primary cutaneous and metastatic melanomas across anatomical sites (cutaneous, visceral and lymph node metastases) compared with healthy volunteer skin, by integrating bulk RNA-seq (TCGA-SKCM) with normal skin (TARGET-GTEx database) datasets (Supplementary Table 1).

**Fig. 2:**
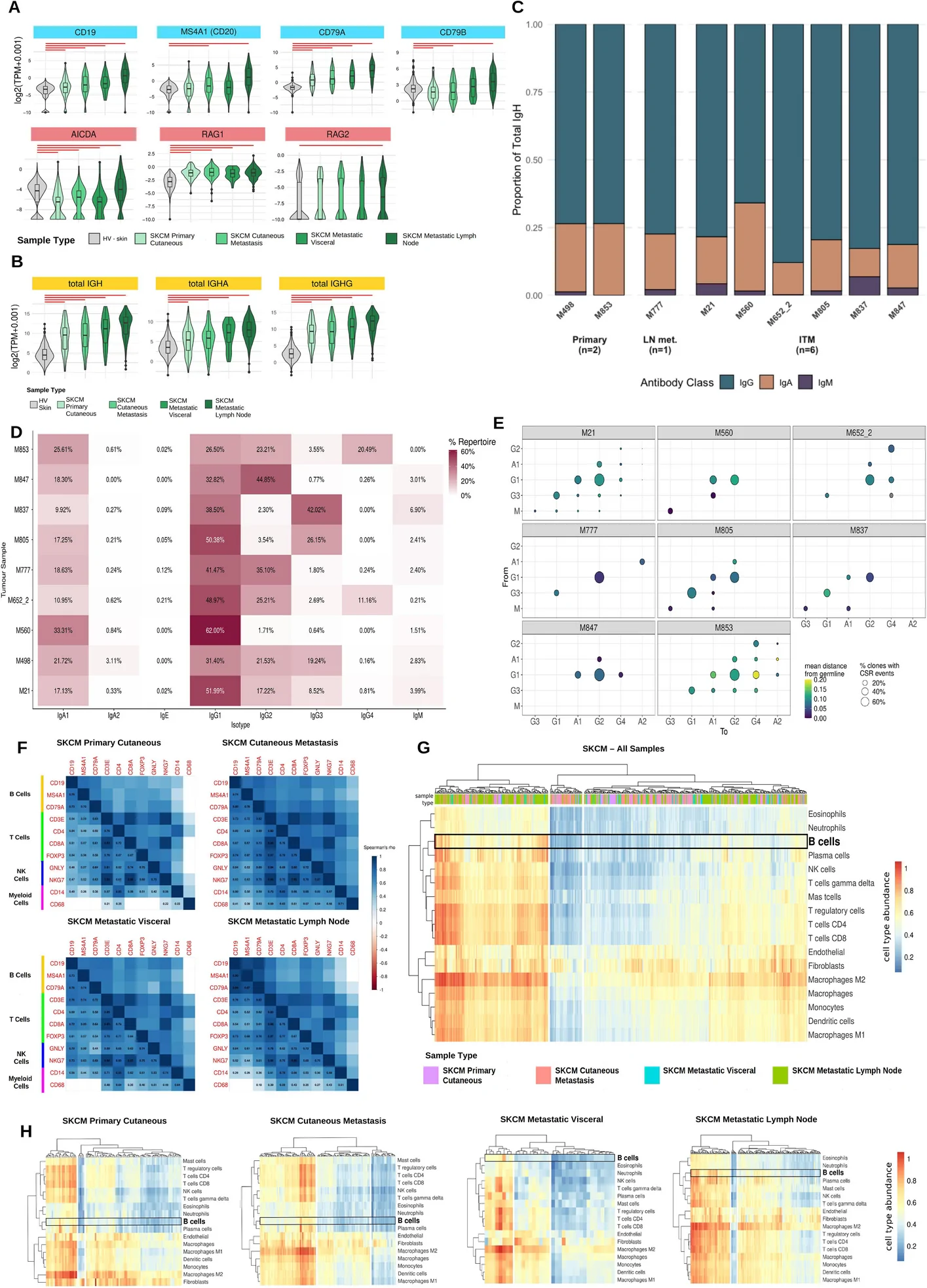
Bulk RNA sequencing and long-read sequencing data analysis shows expression of B cell markers and genes associated with class-switch recombination, somatic hypermutation, VDJ recombination and immunoglobulin expression in human melanoma tumors and B cell infiltration within immune rich tumors. **A** Gene expression studied in melanoma tissue from TCGA-SKCM dataset (*n* = 451) and healthy tissue from TARGET GTEx (*n* = 556) shows expression for the B cell genes and class-switch recombination associated genes AICDA and RAG1/2 in SKCM. n represents number of patients. **B** Comparison of total heavy chain antibody genes across progression stages of melanoma compared to healthy skin. Red lines indicate significant differences in the expression of each gene against healthy skin, after FDR correction (padj ≤ 0.05). **C** Proportion of antibody class relative to total IgH in melanoma samples (*n* = 9). **D** Heatmap visualizes the percentage of antibody isotypes in melanoma samples (*n* = 9). **E** Dot plots show class-switch recombination in melanoma samples towards IgG1/2/4 and IgA1 (*n* = 8). Positive correlation of B cells with other immune cells, and the association of B cell infiltration with “medium” or “hot” immunogenic tumor types. **F** Spearman’s correlation of pan-B cell markers (CD19, MS4A1 (CD20), CD79A) with transcriptomic markers for T cells (CD3E, CD4, CD8A, FOXP3), NK cells, (GNLY, NKG7) and myeloid populations (CD14, CD68) across the four melanoma progression stages shows a strong positive correlation (rho > 0.5) of T cells and NK cells with B cell markers. Numbers represent FDR-corrected p values, and only FDR < = 0.05 are shown. **G** Immune cell types derived from ConsesusTME deconvolution shows clear separation of melanoma samples into “hot”, “medium” and “cold” tumors based on overall immune infiltration, with “medium” and “hot” tumors enriched in B cells. **H** Melanoma samples can be split into the three immunogenic categories across all progression stages. B cells are enriched in the “medium” and “hot” tumors across progression stages. Columns represent samples, rows represent cell types/composite gene signatures available in ConsensusTME

Canonical B cell marker (*CD19*, *MS4A1 (CD20), CD79A, CD79B*) expression ranked highly in melanoma (SKCM) compared to other cancer types (Supplementary Fig. 1 A). *CD19* and *CD79A* were enriched in all melanoma samples compared with normal skin, while *CD20 was* significantly enriched in cutaneous and lymph node metastases (Fig. 2A). Somatic-hypermutation (*AICDA*) and class-switch recombination (*RAG1/2*) enzyme expression were detected across sites; *RAG1 was* upregulated in all melanoma samples compared to healthy skin, denoting dynamic VDJ-recombination and BCR diversification in situ (Fig. 2A).

Analysis of immunoglobulin heavy chain (*IGH*) gene expression in SKCM showed the greatest *IGHA1/IGHA* and *IGHG1/IGHG* ratios compared to other cancers (Supplementary Fig. 1B). Consistent with higher B cell infiltration across cutaneous to visceral and lymph node metastases, total *IGH, IGHA and IGHG* class-switched transcripts were enriched in all melanoma sites compared to normal skin (Fig. 2B). In concordance, long-read antibody transcriptomic analysis of human melanoma specimens (*n* = 9, Supplementary Table 1) confirmed that all tumors contained high proportions of IgG and IgA relative to total IgH, with small proportions of IgM (Fig. 2C). In addition, we observe that IgG1 and IgA1, followed by IgG2/3/4, contributed the greatest proportion of antibodies expressed across samples (Fig. 2D), and that whilst all patients share commonalities such as high frequency of CSR from IgG1 to IgG2 and IgG1 to IgA1, they overall show different CSR trajectories (*n* = 8) (Fig. 2E).

Taken together, bulk RNA-seq and antibody long-read transcriptomic analyses point to recurrent class-switched B cells and antibody IgG/IgA-biased isotype distributions that are most likely expressed across primary and metastatic melanoma samples examined.

### B cell infiltration aligns with T cell and NK cell densities in immune-rich “hot” primary and metastatic melanomas

We next investigated B cell infiltration density in relation to other immune cell populations (Spearman’s correlation) in melanoma lesions: B cells (*CD19, MS4A1 (CD20), CD79A*), T cells (*CD3E, CD4, CD8A, FOXP3*), NK cells, (*GNLY, NKG7*) and myeloid (*CD14, CD68*) populations across tumor sites. B cell markers show strong positive correlation with T cells and NK cells (rho > 0.5), and moderate correlation with myeloid cells (*CD14*) (0.2 < rho < 0.4) in primary cutaneous melanomas and lymph node metastases and a stronger correlation (rho > 0.5) in cutaneous and visceral metastases. *CD68* shows low correlation only in cutaneous metastases with B cells (Fig. 2F).

To further understand immune composition across melanoma primary and metastatic sites, we used the ConsesusTME tool to deconvolute bulk transcriptomic data, with enrichment normalized to allow comparison of a cell population across all samples. Deconvolution of transcriptomic data from all samples was conducted to understand which melanoma category was most enriched in immune cell populations (Fig. 2G). Unsupervised hierarchical clustering of the normalized enrichment score showed, as expected, lymph node metastases with the highest immune enrichment. We observed a clear separation of samples into three sets: “hot”, “medium” and “cold” tumors. This segregation was based on the top level dendrogram branching and reflects the overall immune infiltration. “Hot” tumors showed high levels of immune infiltration while “cold” show low levels. Interestingly, cells of myeloid lineage (macrophages/monocytes) were present in high levels in almost all tumors irrespective of immune score. Overall, B cell infiltration is associated with “medium” or “hot” tumor types across all samples (Fig. 2G), and tumor sites (Fig. 2H).

B cell infiltration aligns with T and NK cells, while macrophage infiltration is observed in all tumors, independently of overall immune cell infiltration and density.

### Memory, activated-IFN and memory-IFN B cells in melanoma pre- and post-treatment with immunotherapy revealed by single-cell RNA-seq analyses

Based on evidence of active class-switching across anatomical sites, we explored B cell differentiation trajectories in two public scRNA-seq datasets of melanoma samples prior to and following checkpoint inhibitor immunotherapy.

One dataset included 19 tumor samples (GSE123139) prior to (*n* = 12) and following (*n* = 7, of which 2 on-treatment samples) treatment with checkpoint inhibitor antibodies (anti-PD1, or anti-PD1 + anti-CTLA4 combination) (Supplementary Table 2) [28].

**Fig. 3:**
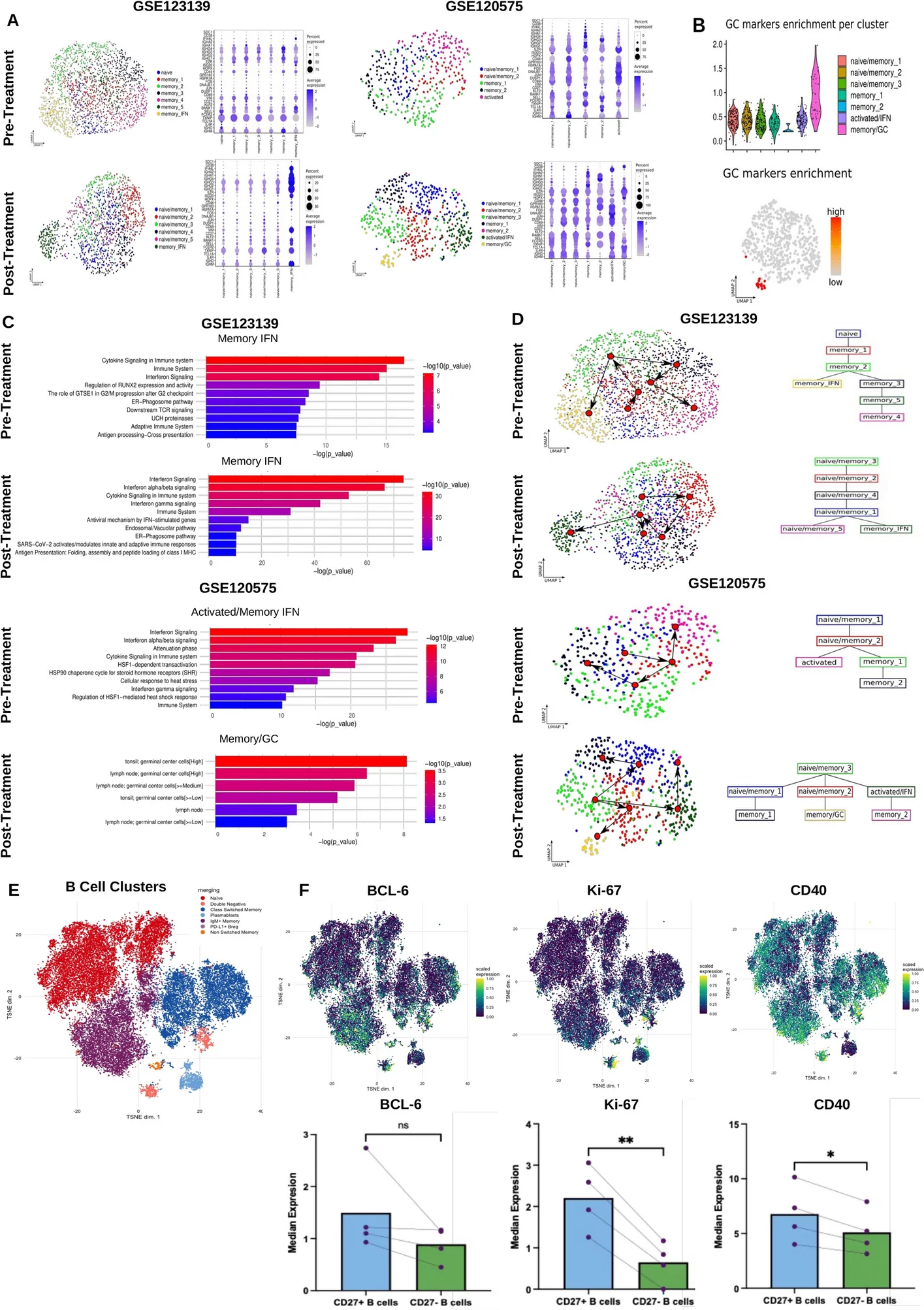
Single-cell RNA sequencing and CyTOF analysis reveals B cell populations and B cell differentiation in pre- and post-treatment samples from melanoma patients. **A** UMAP plots show the B cell subtypes in the single-cell RNA sequencing datasets (GSE123139 and GSE120575) in pre-(*n* = 7 and *n* = 5, respectively) and post-treated (*n* = 15 and *n* = 18, respectively) patient melanoma samples. Dot plots demonstrate the key markers used to annotate each B cell subset. **B** Germinal Centre (GC) signature (King et al.) enrichment score demonstrates high GC enrichment in memory/GC B cells from post-treated cells population in the GSE120575 dataset. **C** Significant DEGs from B cell subsets analyzed by gene over-representation analysis with Reactome and Protein Atlas pathways show pathways associated with each B cell subset. **D** Pseudotime analysis defines the relationship between B cell clusters to capture cell progression from naive to memory, IFN-memory and activated B cell subtypes. **E**, **F** Immunophenotyping of B cells by CyTOF in four melanoma tumors. FlowSOM clustering was used to generate eight CD27 + B cell subsets from the tumors of four melanoma patients, visualized with TSNE. GC, proliferative and cellular activation markers (BCL6, Ki67, CD40) were found to be expressed by CD27 + B cells in the tumor. *p < 0.05; **p < 0.01

Only B cell clusters were selected for downstream analysis, allowing selection of 7 samples pre-treatment and 5 samples post-treatment. In pre-treatment samples, we annotated seven clusters: one naive (*IGHM, IGHD, IL4R, TCL1A*), five distinct memory (*IGHG1/2/3/4, IGHA1, CD27*) and one transcriptionally distinct memory-IFN B cell cluster (*GBP4, GBP1, IFITM1, IFI44L*) (Fig. 3A, Supplementary Fig. 2 A and 3 A). Post-treatment, we detected six clusters: five naive/memory subsets based on mixed naive (*IGHM, IGHD, IL4R, TCL1A*) and memory (*IGHG1/2/3/4, IGHA1, CD27*) marker expression, and one memory-IFN B cell subset (*IFIT3, IFI44L, IFIT2, IFIT1*) (Fig. 3A, Supplementary Fig. 2 A and 3B).

The second dataset GSE120575 [33] contained 43 samples from 32 patients, collected at baseline (*n* = 19) and post-treatment (*n* = 24) with anti-PD1/anti-CTLA4 (Supplementary Table 3).

Only B cell clusters were selected for downstream analysis. B cells were detected in a subset of samples, allowing selection of 15 pre-treatment and 18 post-treatment samples. In the pre-treatment cohort, two clusters were mixed naive/memory, two memory clusters and another activated population (*CD69, JUN, DUSP1* associated with activation, B cell receptor (BCR) formation and downstream signaling). Seven clusters were identified post-treatment: three mixed naive/memory B cell subsets, two memory B cell clusters, a distinct activated-IFN cluster with mixed activation (*IFI44L, GBP4*) (Fig. 3A, Supplementary Fig. 2B and 3 C), BCR formation (*CD69, JUN, DUSP1*) and a memory/GC-like B cell cluster, for which module scoring confirms enrichment of previously reported GC signature [25] (Fig. 3A, 3B). Further investigation of differentially expressed genes using gene over-representation analysis, confirmed activation of cytokine, specifically IFN, signaling pathways pre- as well as post-treatment, and GC pathway activation post-treatment (Fig. 3C).

To track B cell differentiation dynamics in the TME, we performed unsupervised pseudotime developmental trajectory analysis using the Slingshot package, whereby the starting cluster was not specified. In all cases, a naive or naive/memory cluster was identified as the starting cluster [37]. Progression from naive to memory, to memory-IFN and activated-IFN B cell populations was captured across both scRNA-seq datasets. These trajectories highlighted predicted transitions in differentiation complexity from naive or naive/memory B cells toward more complex memory or activated states. In almost all cases, and observed in both pre- and post-treatment tumors, memory-IFN B cells were either present, retained post-treatment and found as predicted terminal states in differentiation trajectories or activated-IFN B cells are observed post-treatment (Fig. 3D). These findings point to an inflammation associated B cell evolution with activated-IFN and memory-IFN B cells identified often as predicted endpoint states in differentiation trajectories, prior to, and post-immunotherapy.

The presence of terminally differentiated B cells with an IFN phenotype was of particular interest. We therefore further explored the expression of IFN-associated genes within the scRNA-seq datasets. These cells displayed expression of IGHD and CD27, together with canonical interferon-stimulated genes, including GBP1, GBP2, GBP4, IFITM1, IFIT1, IFIT2, IFIT3, and MX1 (Supplementary Fig. 3 A, 3B, 3 C) consistent with an activated IFN-response state [20]. To validate this observation in an independent dataset, we analyzed flow cytometry-sorted and pooled B cells from melanoma tumors (n = 7) alongside matched CD45- cells from the same specimens (Supplementary Fig. 4 A). Tumor-derived B cells demonstrated higher expression of multiple IFN-response genes (Supplementary Fig. 4B), and gene set variation analysis (GSVA) revealed enrichment of IFN-alpha and IFN-beta response signatures in B cells relative to CD45- cells (Supplementary Fig. 4 C), further supporting the presence of an IFN-associated transcriptional program in intratumoral B cells.

To explore differentiated B cell populations at the protein level, we conducted high-dimensional (CyTOF) analyses of B cells from four melanoma samples (Supplementary Tables 4 and 5). FlowSOM clustering identified seven B cell canonical subsets (RRID:SCR_016899) (Fig. 3E) (relative abundances per tumor sample in Supplementary Fig. 5 A). Stratification by CD27 (memory B cells) and CD27- expression (Supplementary Fig. 5B) revealed significant enrichment of the proliferation marker Ki-67 and the activation and class-switching co-stimulatory CD40 on memory B cells. Both CD27 + and CD27- B cells expressed the GC marker BCL-6 (Fig. 3F). The CD27 + B cell compartment showed expression of BCL-6, Ki-67 and CXCR5, consistent with proliferative and GC-associated phenotypes (Supplementary Fig. 5 C).

Across datasets, melanoma-infiltrating B cells exhibited differentiation trajectories, terminating in memory, IFN-associated predicted terminal states that persist after immunotherapy. CyTOF analyses independently confirm proliferative, GC-like, memory, CD40-expressing and class-switched B cell populations, supporting the presence of conserved inflammatory B cell states identified across transcriptomic datasets.

### Memory and IFN-rich B cell subsets preferentially express IgA1, IgA2, IgG4 and IgM/IgD B cells are retained or induced in post-treatment melanoma

We next evaluated antibody isotype expression pre- and post-immunotherapy in single B cell subsets from the two scRNA-seq datasets prior to and post immunotherapy.

**Fig. 4:**
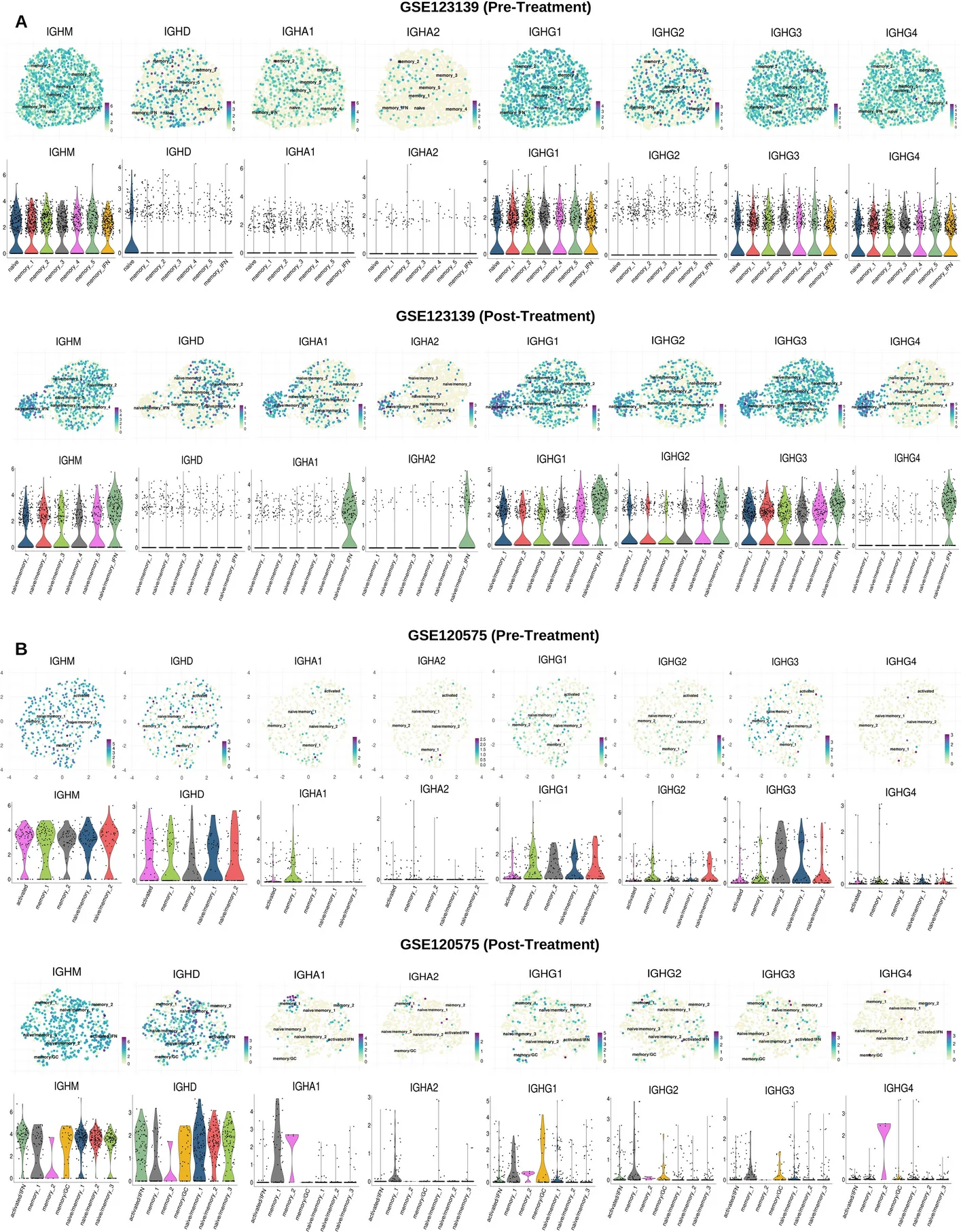
Antibody repertoire genes projected on single-cell RNA sequencing UMAP and violin plots across pre- and post-treated tumor cohorts. Single B cells were analyzed from GSE123139 and GSE120575 datasets. **A** Violin plots show IgA1, IgA2 and IgG4 antibody isotypes are retained in memory IFN B cells post-treatment in the GSE123139 dataset. **B** IgA1, IgA2, IgG1 and IgG4 antibody isotypes are retained in memory B cell clusters, and IgG1 is enriched in GC-like memory B cells post-treatment in the GSE120575 dataset

Both cohorts, prior to immunotherapy, showed wide antibody isotype distribution, prominently IgM, IgG1, IgG3, across B cell subsets. Post-treatment samples showed no additional class-switching in B cell populations, however both datasets showed retention or induction of IgM and IgD, suggesting likely de novo humoral immune response initiation/B cell recruitment. In GSE123139, the memory-IFN B cell cluster post-treatment is the only subset to express IgA1 and IgA2 and to retain IgG4 expression (Fig. 4A).

In GSE120575 post-treatment, memory_1 cluster was enriched in IgA1 and IgA2 and retained IgG1 expression, and memory_2 cluster were enriched in IgA2 and IgG4 post-treatment, suggesting favoring of alternatively-activated isotypes. Furthermore, the newly-induced activated-IFN cluster was not class switched as expression of IgM and IgD was retained, and the GC-like memory B cell cluster post-treatment was IgG1 + and to a lesser extent IgG2/IgG3 + (Fig. 4B).

Overall, class-switched B cells were retained in tumors post-treatment, and IgA1, IgA2 and IgG4 were preferentially expressed by memory and IFN-rich B cell subsets after immunotherapy, suggesting a drive towards alternatively activated responses. Furthermore, post-treatment retention or induction of IgM/IgD expression and a new IgM + IgD + activated-IFN subset suggest de novo B cell initiation.

### Spatial transcriptomic analyses support B cell infiltration in immune “hot” tumors and assembly in active BCR and antibody differentiation sites, GC and TLS areas

**Fig. 5:**
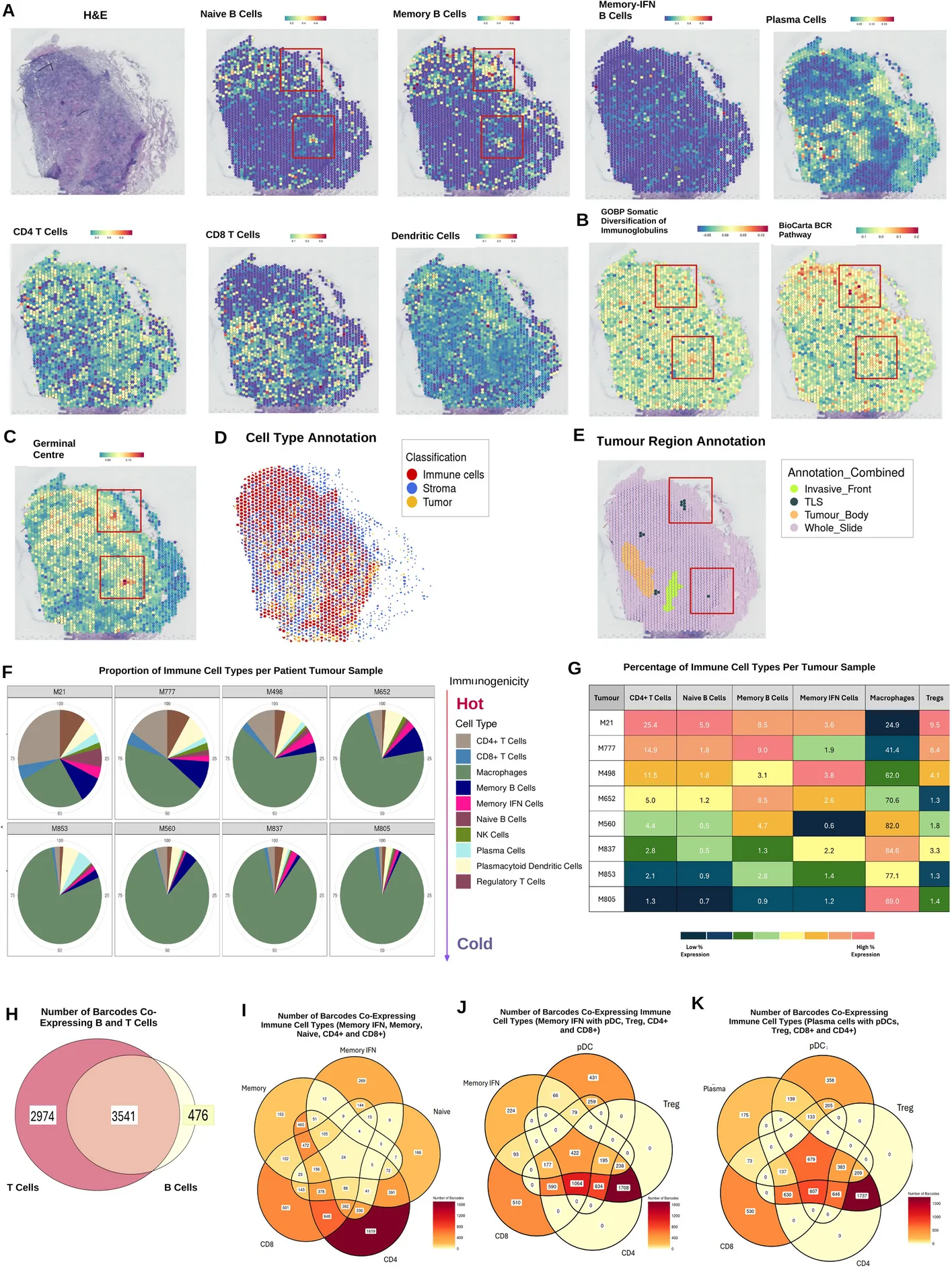
Spatial transcriptomic analysis of B cell localization alongside T cell, germinal center and lymphoid features in melanoma lesions. **A** Representative images (M21) of deconvoluted spatial transcriptomic melanoma tumor slices show infiltration of naive, memory and memory-IFN B cells, CD4 + and CD8 + T cells, plasma cells, and dendritic cells, alongside plasmablast signature enrichment. **B** High enrichment scores were revealed when analyzing the expression of the gene sets ‘somatic differentiation of immunoglobulins’ and ‘BCR signaling pathway’ from MsigDB. **C** Enrichment of a germinal-center (GC) signature reveals high GC enrichment score in both B cell localization zones. **D**-**E** Histological annotation in representative tumor slice shows cell type annotation including immune, tumor and stromal cells (**D**) and tumor regions including (**E**) invasive front, tertiary lymphoid structure (TLS) and tumor body. **F** Quantification of B cell density visualized by pie charts shows the proportion of immune cell populations per patient tumor sample. Tumors can be stratified into immunogenic “hot” and “cold” tumors, with greater B cell infiltration observed in “hot” tumors. The proportion of macrophages was greatest in samples M805, M837 and M560 (89%, 84.6% and 82%), in which the percentage of infiltrating immune cells was relatively low: memory B cells (0.9%, 1.3% and 4.7%), naive B cells (0.7%, 0.5% and 0.5%), CD4 + T cells (1.3%, 2.8% and 4.4%) and CD8 + T cells (1.8%, 1.3% and 0.7%), respectively. (H) Venn diagram shows the number of barcodes co-expressing B and T cell populations across all tumor samples, with 88% of B cells co-localizing with T cells. **I**, **J**, **K** Venn diagrams show co-localization of specific B and T cell subsets across all melanoma samples, whereby (**I**) greater co-localization of memory-IFN B cells with pDCs, Tregs and CD4 +/CD8 + T cells are found compared to (**J**) with memory and naive B cells and CD4 +/CD8 + T cells. (**K**) Venn diagram shows high co-localization of plasma cells with pDCs, Tregs and CD4 +/CD8 + T cells

To investigate the localization and lymphoid assembly of B cell subsets within the TME we conducted spatial transcriptomic analyses of 8 melanoma samples (Supplementary Table 6), and at different lesion depths to assess immune infiltrate heterogeneity (representative tumor images M21: Fig. 5A-E and M498 (Supplementary Fig. 6A-E). Deconvolution was performed using the annotated single-cell dataset as reference. Pan-B cell markers (*CD19*, *CD79A*, *CD79B*, and *MS4A1*) were also mapped onto spatial samples (Supplementary Fig. 6).

B cell localization (examples in red boxes) of memory and memory-IFN B cells were adjacent to CD8 + T cells, a prerequisite for GC-like morphology (representative tumors M21, M498, Fig. 5A, Supplementary Fig. 7 A). B cell-positive localization areas showed high enrichment scores for ‘somatic differentiation of immunoglobulins’ and ‘BCR signaling pathway’ gene set expression (MsigDB) (Fig. 5B, Supplementary Fig. 7B), denoting humoral activation. These features were confirmed by detection of high GC immune enrichment score in B cell localization areas (Fig. 5C, Supplementary Fig. 7 C).

We next mapped transcriptomic data onto histopathological annotation to identify the distribution of immune, stroma and tumor cells (Fig. 5D, Supplementary Fig. 7D), alongside specific tumor regions including the tumor body, tumor invasive front and TLS (Fig. 5E, Supplementary Fig. 7E). These revealed several TLS sites aligning with memory B cell clusters and high GC signature enrichment (red boxes) showing both early/immature (no GC gene signature) and late/mature TLS within the tumor, as defined by the presence of GC gene signatures [18].

These data confirm spatial association of B cells in immune “hot”, BCR signaling pathway-rich and somatic antibody differentiation-rich areas, assembly in GC regions and in TLS.

### Memory, memory-IFN and naïve B cell subsets localize with CD4 +, CD8 + T cells and Tregs and align with enriched GC signatures within TLS

We next quantified B cell subset localization in tumor lesions by spatial transcriptomic and QuPath analyses as previously reported [29]. The overall density of tumor-infiltrating immune cells varied between tumor samples. For instance, M21 featured high levels of diverse immune cell subsets (58.8% of total cells), including memory, memory-IFN B cells and plasma cells, while other tumors (M853, M837) with high immune cell infiltration counts (28.1% and 26.6%, respectively) showed macrophages representing the majority of the immune cell infiltrate (Supplementary Fig. 8 A).

Consistent with normalized enrichment scores in bulk RNA-seq datasets (Fig. 2F-G), spatial transcriptomic analyses confirmed that all tumors were infiltrated by macrophages (range 24.9–89%), while some (M21, M777, M498, M652) showed prominent CD4 + T cell infiltration (25.4, 14.9 11.5, 5%, respectively), and B cell populations including memory (8.5, 1.9, 3.1, 8.5%) and naive (5.9, 1.8, 1.8, 1.2%) subsets, were are considered immunogenic “hot” (Fig. 5F, 5G).

Within B cells, the proportion of memory-IFN was greatest in the immunogenic “hot” (M498: 3.8%, M21: 3.6%, M652: 2.6%) tumors, while a relatively high proportion of memory-IFN B cells were still present in M837 (2.2%), featuring high macrophage infiltration, low infiltration of other B cell populations, and greatest Treg infiltration (3.4%) compared to immunogenically cold (M853, M805, M560) tumors (Tregs ranging from 1.3–1.8%). This is compared to Treg infiltration in the immunogenic (M21: 9.5%, M777: 8.4%, M498: 4.1%) tumors (Fig. 5G). Immune cell infiltration between slices of the same tumor sample appeared to be largely consistent (Supplementary Fig. 9).

We next sought to understand where B cells are localized in relation to other immune cell populations and tumor regions including the TLS. We examined 4,017 B cell-containing spots across eight specimens. We found a high level of co-localization, with 88% (3,541 of 4,017) of all B cell + spots in tumors co-localized with T cell + spots (Fig. 5H). Cell type co-localization of the B cell subsets (memory-IFN, memory and naive B cells) with CD4 + and CD8 + T cells showed greatest co-localization frequency (472 spots) for CD4 + and CD8 + T cells with memory B cells, followed by CD4 + T cells with memory B cells alone (460). Naive B cells also highly co-localize with both CD4 + and CD8 + T cells (378), and with CD4 + T cells only (391). There were fewer spots containing the co-localization of naive B cells and CD8 + T cells only (143). Memory-IFN B cells mostly co-localize with both CD4 + and CD8 + T cells together (382) and with CD4 + T cells alone (336). Overall, high co-localization of B cell subtypes with CD4 + T cells reflected B and T cell interactions and antigen presentation [18] (Fig. 5I).

Next, co-localization of memory-IFN B cells with pDCs, Tregs and CD4 + CD8 + T cells also showed high co-localization of memory-IFN B cells together with CD4 +, CD8 + T cells, DCs and Tregs (422 spots), and with only CD4 + T cells and Tregs (238 spots), with CD4 + T cells, Tregs and DCs (195 spots) and with CD4 + and CD8 + T cells only (177 spots) (Fig. 5J). These suggest that memory-IFN B cells follow a pattern similar to that of other B cells in co-localization with T cells.

In the next co-localization analyses we explored the co-localization of plasma cells with Tregs, pDCs, CD4 + and CD8 + T cells. We observed the highest co-localization of plasma cells with all cell types together (CD8 +, CD4 + T cells and Tregs, alongside pDCs—679 spots), followed by CD4 +, Tregs and DCs (383 spots) (Fig. 5K).

Overall, we observe higher co-occurrence of memory IFN, pDC and Treg populations with CD4 + and CD8 + T cells (422 spots) compared to all three B cell subtypes (memory IFN, memory and naive) with CD4 + and CD8 + T cells (24 spots) (Fig. 5I, 5 J).

### B cell interactions with tumor-resident T cells and DCs are enhanced following immunotherapy

Having established significant correlations between B cells and major immune cell populations in the TME in the bulk RNA-seq analysis, alongside co-localization in the spatial transcriptomic samples, we next explored ligand-receptor interactions before and after immunotherapy in the scRNA-seq datasets (R package Liana) [13].

Pre-treatment in GSE123139, B cells engaged in 13 outgoing interactions providing ligands to DCs (7 interactions), CD8 T cells (5 interactions) and regulatory T cells (Tregs) (1 interaction). B cells receive signals from DCs (1) and from plasma cells (1). Post-treatment (GSE123139), B cells sender interactions doubled to 28: with DCs (9 interactions), CD8 T cells (7 interactions), and Tregs (11). Interactions where B cells provided the receptor increased from 2 at pre-treatment to 5 post-treatment: with DCs (3) and monocytes/macrophages (2) (Supplementary Fig. 10 A).

Pre-treatment in GSE120575 [33], B cells provide ligands to monocytes/macrophages (22), DCs (15), Tregs (15), CD8 T cells (12), NK cells (8) and other B cells (6). Interactions where B cells were receiving a ligand come from monocytes/macrophages (29) and DCs (9). Post-treatment, B cell interactions in both receiver and sender roles increased. B cells provided ligands to monocytes/macrophages (21), Tregs (19) and DCs (16). The most interactions of cells providing ligands to B cells were monocytes/macrophages (27), DCs (14) and CD8 T cells (8) (Supplementary Fig. 8 A). Overall, across datasets, post-treatment tumors showed higher numbers of significant interaction pairs between B cells and other infiltrating immune cells (Supplementary Fig. 10B).

Aligned with immune-rich tumor correlations, these findings indicate enhanced post-immunotherapy B cell communication with multiple immune populations, particularly T cells and DCs, through reciprocal ligand–receptor signaling.

### T and B cell co-localization aligns with early and mature TLS in tumors

**Fig. 6:**
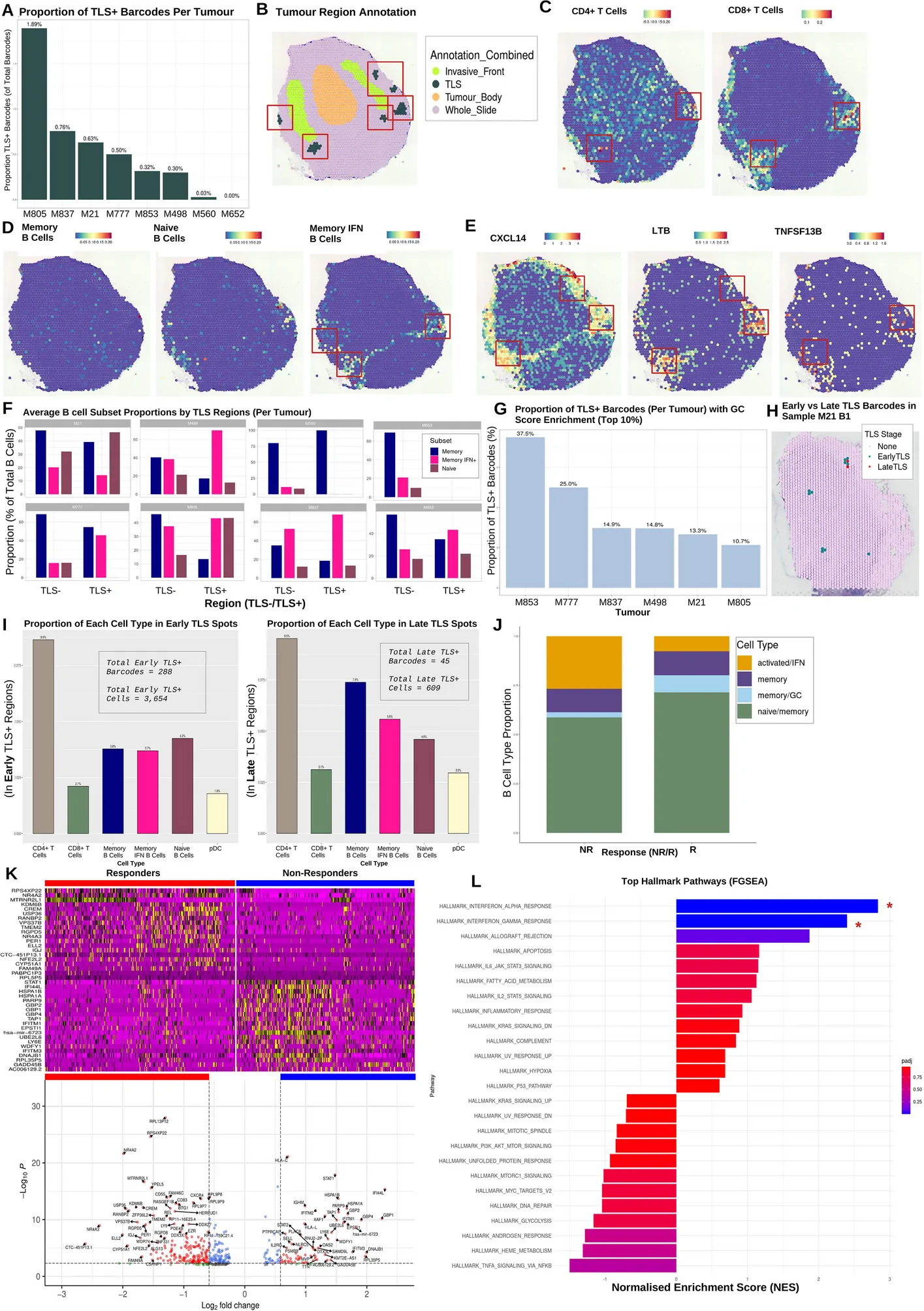
Association of B cell subsets in TLS melanoma lesions by spatial transcriptomic analysis, and the comparison of tumor-resident B cell profiles between responders and non-responders following checkpoint inhibitor immunotherapy. (**A**) Proportion of TLS + barcodes per tumor. (**B**) Annotated M805 tumor with (**C**) localized T cell and (**D**) B cell subsets in TLS + regions. (**E**) The expression of early TLS markers (CXCL14, LTB and TNFSF13B (BAFF)) in the same tumor sample M805. Red boxes highlight localization. (**F**) Specific B cell populations in TLS- versus TLS + regions as a proportion of total B cells. (**G**) TLS + regions with GC enrichment score within the top decile. (**H**) Representative image shows early and late TLS spots in sample M21 B1. (**I**) The proportion of CD4 + and CD8 + T cells, memory, memory-IFN, and naive B cells, and pDC in early (*n* = 288) and late (*n* = 45) TLS spots. Cell type proportions calculated as percentage of each cell type relative to the total number of cells in early (*n* = 3,654 cells) and late (*n* = 609 cells) TLS. (**J**) Proportions of B cell subtypes between total patients and per patient in the non-responder and responder groups. There is a higher proportion of memory/germinal center like B cells in the responders, while there is a higher proportion of memory-IFN producing B cells in the non-responders. (**K**) Differential gene analysis between the B cell profiles of non-responder versus responder patient groups revealed distinctly different transcriptional profiles. Heatmap shows the top 20 significant (padj < = 0.05) DEGs. (L) Fast gene set enrichment analysis (fgsea) calculated the normalized enrichment score (NES) of the non-responder DEGs in hallmark pathways. A significantly positive (padj < = 0.05) NES, denoted by an asterisk, was observed for the IFN-α and IFN-β response pathways

Since high levels of co-localization of B cell populations with T cell subsets may be important for GC reactions and the formation of TLS, we investigated the proportion of TLS + regions in each tumor sample, and their immune cell composition by spatial transcriptomic analyses. Annotated TLS + regions ranged from a proportion of 0–1.89% of all transcriptomic spots per tumor sample (Fig. 6A). Interestingly, while M805 has relatively low overall CD4 + T cell and B cell density, it has the greatest proportion of TLS + regions, featuring highly co-localized B and T cells, rather than wide-spread distribution across the tumor area. This aligns with TLS annotation (Fig. 6B), localization of T (Fig. 6C) and B (Fig. 6D) cell subsets, and expression of early TLS markers (e.g., *CXCL14, LTB, TNFSF13B* (*BAFF*)) [22, 35] (Fig. 6E), while lacking expression of late TLS markers *CD21* (*CD2*) and *CD23* (*FCER2*) [4].

We further investigated the cellular composition of TLS structures across all melanoma samples. Memory B cells and naive B cells made up 6.4% while memory-IFN B cells make up 6% of TLS. Of T cell populations, CD4 + T cells made up 13.2% of TLS, followed by Tregs (8.5%) and CD8 + T cells (3.4%). Interestingly, consistent with published studies, macrophages made up 50.8% of TLS, suggesting their participation in lymphoid assembly, and in immune-mediated tumor promoting or immunosuppressive response [8, 38] (Supplementary Fig. 11 A).

We next sought to understand how the proportion of B cell populations, memory-IFN, memory and naive B cells as a proportion of total B cells, varied inside and outside of TLS regions. Memory-IFN B cells were consistently observed within TLS + regions across all tumor samples except for sample M560, which contained only 1 memory B cell-enriched TLS + spot across all slices. In particular, a greater proportion of memory-IFN B cells were localized within the TLS in 5/7 tumor samples containing TLS regions (Fig. 6F), suggesting that the cells may have an IFN phenotype alongside expression of *IGHD* and *CD27* genes, and undergo early follicular stimulation (*GBP1/2/4, IFITM1, IFIT1/2/3, MX1)* (Supplementary Fig. 3 A, 3B, 3 C) [20]. Melanoma lesions contained immature and mature TLS, the latter defined by the presence of GC gene signatures [4]. We next studied enrichment of GC signature scores as a sign of late TLS in samples containing TLS regions. The proportion of TLS spots containing top 10% of GC enrichment scores ranged from 10.7–37.5% of TLS (Fig. 6G), suggesting the presence of late, mature TLS (Fig. 6H). In addition, these regions showed not only the expression of early TLS markers (*CXCL14, LTB, TNFSF13B* (*BAFF*)) (Supplementary Fig. 11B), but also the expression of secondary follicle structure features indicated by the expression of *CD21* (*CR2*), *CD23* (*FCER2*) and *BCL6* (Supplementary Fig. 11 C) and GC signature, indicating mature TLS [4]. We further investigated the proportions of B cell subtypes (memory, memory-IFN and naive B cells), CD4 + and CD8 + T cells, and DCs relative to the total number of cells within early (n = 3,654 cells) and late (n = 609 cells) TLS areas. We observe higher overall percentage of all immune cell types in the late TLS areas. Notably, the percentage of memory and of memory-IFN B cells was higher in late TLS (7.4 and 5.8%) versus early TLS (3.8 and 3.7%) (Fig. 6I).

Overall, memory, memory-IFN and naïve B cells were located across “hot” tumors showing high levels of co-localization with CD4 +, CD8 + T cells and Tregs, and memory, memory-IFN B cell and T cell aggregation aligned with both early and mature TLS detected by histopathological annotation.

### Differential tumor-resident B cell profiles in responders and non-responders following checkpoint inhibitor immunotherapy

We next sought to explore associations between B cell states and immunotherapy outcome. Post-treatment samples (*n* = 16) in the GSE120575 dataset [33] containing the previously identified B cell subtypes were stratified into non-responder and responder groups (UMAP projections, Supplementary Fig. 12 A). A relative enrichment of activated-IFN B cells along with lower levels of naïve/memory B cells were seen in non-responders (Fig. 6J). However, this pattern was not uniform across patients and was driven predominantly by a subset of tumors, most prominently in patient 13 treated with anti-CTLA4 + anti-PD1 combination (termed as non-responder with a mixed response) (Supplementary Fig. 12B). A higher proportion of naïve memory B cells and GC-like memory B cells were detected in the responder cohort, most prominent in patient 4, treated with anti-CTLA4 + anti-PD1 combination.

We next evaluated differentially-expressed genes (DEGs) between responder and non-responder patients using FindAllMarkers, focusing on the top 20 most significantly altered markers (average log2 fold change) (Fig. 6K). Responder group B cells showed higher expression of genes associated with antigen stimulation, adaptive immunity and cell survival, including *NR4A2, NR4A3, MTRNR2L1* and *CREM* [5, 30, 42]. Non-responder B cells showed increased expression of interferon-associated genes including *STAT1, GBP1, GBP2, and GBP4* [2, 14] (Fig. 6K, Supplementary Table 7).

Fast gene set enrichment analysis (fgsea) to evaluate the normalized enrichment score (NES) of the non-responder DEGs in hallmark pathways, showed significantly positive (padj < = 0.05) association with upregulated IFN-α and IFN-β response pathways in non-responders (Fig. 6L), consistent with increased interferon-associated transcriptional activity in B cells in this cohort.

Cell–cell communication analysis (GSE120575) [33] in the scRNA-seq datasets (R package Liana) [13] to infer ligand–receptor interactions was performed separately in responders and non-responders, and top-ranked interactions identified B cell communication networks (Supplementary Fig. 13). Responder tumors showed interactions linked to antigen presentation and immune coordination, including *HLA-II/CD4, IL1B–IL1R2, CCL5* and complement-associated signalling. Non-responder tumors were relatively enriched for inflammatory myeloid-associated signals (*S100A8/S100A9*, *CD14*), Fc receptor interactions and tissue-remodelling pathways. Together, these findings suggest variation in B cell states and communication networks across groups, with IFN-associated programs observed more prominently in a subset of non-responder tumors.

These findings point towards distinct B cell states and interaction networks which may be associated with immunotherapy outcomes in this cohort, with responder tumors showing features of adaptive immune coordination and a subset of non-responder tumors displaying enhanced interferon-associated and myeloid-linked inflammatory signatures.

## Discussion

We conducted in-depth characterization of melanomas by bulk, single-cell and spatial transcriptomic analyses to evaluate B cell differentiation trajectories, isotype distribution and spatial organization, and associations with immunotherapy response. B cell density aligns with T cells and DCs in immune-rich tumors, BCR-active profiles and evolution to terminally differentiated, class-switched, and atypical IFN-rich states. We show skewed antibody isotype profiles and IFN-driven B cell signaling pre- and post-treatment with checkpoint inhibitor immunotherapy.

Canonical B cell markers alongside CSR and SHM associated genes were enriched in melanoma across primary and metastatic cutaneous lesions, lymph node and visceral metastasis sites, compared to healthy skin. Bulk RNA-seq cell type deconvolution further supported active class-switching drivers and strong correlation of B cell density with immune rich tumors across disease sites, highlighting a dynamic and evolving humoral immune response. Tumor-infiltrating B cells demonstrate a combination of pro- or anti-inflammatory trajectories. Single-cell annotation of melanoma-associated B cells across two scRNA-seq datasets revealed mixed memory, activated, memory-IFN subsets and GC-like memory B cells in remaining tumors post-treatment, suggesting both terminally differentiated and de novo humoral response induction. Although the relative abundance and transcriptional granularity of individual clusters differed between datasets, reflecting biological and technical heterogeneity, the recurrence of these differentiation trajectories supports the presence of these populations across these two independent melanoma cohorts.

Spatial transcriptomic analyses also support localization of memory B cells surrounded by CD4 +, CD8 + T cells and enrichment of GC signatures, further indicating that B cells are capable of induction of adaptive immunity in situ, previously associated with improved patient outcomes [10, 17] ) Cipponi et al., 2012). IFN-rich B cells localized within TLS and IFN-rich B cell signatures were most likely to express non-class switched immunoglobulin (IgM +/IgD +) or retain expression of immunoregulatory isotypes such as IgG4; IFN-rich B cells, genes and pathways post-treatment with immunotherapy were more prevalent in remaining tumors of non-responders. These features link this IFN-rich compartment with less immune active, alternatively-activated responses.

Similarly to bulk RNA-seq deconvolution, we observe variable immune cell infiltrate densities between melanoma lesions by spatial transcriptomic deconvolution analyses. While macrophage infiltration is common across tumor samples, a subset of tumors displays an immunogenic profile characterized by B and T cell, DC, and plasma cell infiltration. Topologically, CD4 + and CD8 + T cells were found alongside memory, naive and memory-IFN B cells within TLS + regions, which include macrophages. This is consistent with reports of activated *CD169* + macrophages spatially associated within TLS, and with regulatory T and B cell populations in mature TLS in breast cancer [8]. Associations with greater overall survival (OS) in acral melanoma have been ascribed to the presence of TLS whereby *CD68* + macrophages were identified in TLS with proximity to CD8 + T cells [38]. Besides macrophages, focused analysis of B cell populations within TLS regions relative to the tumor area reveal TLS-associated memory-IFN B cells, which may indicate a mixed phenotype population which has undergone early follicular stimulation [20].

Antibody isotype distribution analyses across B cells supported a bias towards class-switched phenotypes indicated by bulk RNA-seq sequencing analyses revealing greater total *IGHA* and *IGHG* expression in melanomas across cutaneous and metastatic sites compared to normal skin. These findings also align with in situ expression of enzymes (*AID, RAG1/2*) responsible for CSR and SHM and consistent with our previously-reported skewed local B cell clonal expansion profiles [12]. Together, these also point to class-switched B cell bias and distinct expanded isotype expression signatures across primary and metastatic melanomas.

We observed greater IgA1- and IgA2- and IgG4-expressing B cell subsets post-treatment. These are consistent with previous evidence of alternatively-activated IgG4 and IgA isotypes favored in melanoma [23] and of plasma cell–rich enriched IgA-expressing immune infiltrates in primary melanoma, occurring rarely but mostly observed in older patients, and associated with adverse histopathological features (increased Breslow thickness and ulceration) and worse melanoma-specific survival [7, 17]. Together, these findings support the concept that humoral responses in melanoma can become skewed to adopt “alternatively activated” rather than classical immune response features. In this context, IgG4 and IgA are generally considered less-effective mediators of classical antitumor effector functions relative to IgG1. IgG4 exhibits limited Fcγ receptor engagement and may competitively interfere with IgG1-mediated antibody-dependent cellular cytotoxicity, while both IgG4 and IgA show reduced capacity for complement activation [23, 24]. Within inflammatory tumor microenvironments, these isotypes have also been associated with immunoregulatory immune programmes, raising the possibility that this skewing may contribute to attenuation of effective antitumor immunity. Consistent with this, our findings point to IFN-rich, IgG4/IgA-biased B cell trajectories associated with immunotherapy resistance in melanoma, highlighting divergence in B cell functional states [7, 17]. Here, retention of IgG4 + class-switched memory-IFN B cells post-treatment might align with established roles for IgG4 in anti-inflammatory responses, immune evasion, chronic inflammation and autoimmunity [34]. Furthermore, retention of immunoregulatory IgG4 expression by IFN-rich B cells and induction of IgM + IgD + IFN-rich B cell subsets following immunotherapy, together with early signs of these signatures in tumors from non-responders, suggest atypical inflammatory programmes that may contribute to immune evasion and treatment resistance.

IFN-expressing or IFN-programmed B cells induced by inflammatory cytokines such as IL-12, IL-18, and IFN-α/β, TLR ligands, and chronic antigen exposure have been identified in disease contexts such as autoimmunity, chronic infection, and cancer including melanoma. IFN-driven B cell states often observed among plasmablast, GC-like, or atypical memory populations, share transcriptional features with chronic inflammatory and autoimmune responses [1, 12, 40, 43]. In this context, the IFN-rich B cell populations identified here in our study may reflect prolonged antigenic stimulation and ongoing type I IFN signalling within the TME. Given the established role of type I IFNs in cancer immunoediting, such populations could represent either sustained antitumor immune activation or chronic inflammatory adaptation, depending on tumor context and disease stage. It is possible that type I IFN signalling may initially support antigen presentation, T cell priming and TLS-associated humoral responses [19] Cabrita et al., Nature, [10]. However, prolonged exposure during immunoediting could also favour divergence towards immunoregulatory or alternatively activated B cell states. Our data do not distinguish whether IFN-rich B cells actively contribute to resistance or primarily mark a broader, persistent inflammatory environment associated with less effective immune responses [7]. Early findings of relative enrichment of these signatures in non-responders in our dataset raises the possibility that persistent IFN-associated B cell states may accompany less effective immune responses to immunotherapy, although larger studies will be required to define their functional significance.

Early findings in our study may point to distinct B cell profiles in tumors from responder and non-responder groups to immunotherapy. However, patterns were not uniform across all within these groups, highlighting substantial inter-patient heterogeneity. Although our analyses were limited by the relatively small sizes of the CyTOF, scRNA-seq, spatial transcriptomic and long-read antibody sequencing cohorts, the convergence of complementary molecular, spatial and immunoglobulin repertoire evidence supports ongoing B cell differentiation and class-switching dynamics in melanoma and within immunotherapy. Additionally, the spatial transcriptomic and long-read sequencing cohorts comprised a limited number of heterogeneous melanoma specimens spanning primary tumors, cutaneous in-transit metastases and lymph node metastases. While the major humoral immune features identified, including IgG/IgA-skewed isotype usage, TLS-associated B cell populations and memory-IFN B cell states, were observed across samples, larger cohorts will be required to determine the contribution of tumor site-, stage- and treatment-specific effects.

Furthermore, the two scRNA-seq datasets analyzed were generated from independent patient cohorts with differing clinical characteristics, treatment exposures, and sequencing parameters, contributing to inter-dataset variability in cluster composition and relative population abundance. Nevertheless, several higher-order biological features were consistently observed across datasets. Both cohorts demonstrated a conserved B cell differentiation trajectory from naïve or naïve/memory populations to more differentiated memory and IFN-associated states. Memory B cell populations were identified across different treatment settings, while IFN-rich populations emerged as transcriptionally distinct differentiation states. In addition, both datasets supported enhanced post-treatment B cell interactions with T cells and dendritic cells. These findings were independently supported by CyTOF and spatial transcriptomic analyses, pointing to activated, memory, GC-like and IFN-associated B cell populations within immune-rich and TLS-associated tumor regions. Together, these observations suggest that key B cell programmes identified here represent conserved features of melanoma-associated humoral immunity. However, larger harmonized cohorts will be required to determine the prevalence, prognostic significance and potential utility of these B cell states as biomarkers for precision immunotherapy.

In summary, actively differentiating and class-switching tumor-resident B cells aligned with immune-rich tumors, feature inflammatory profiles, interact with several immune cells such as T cells and DCs, and are localized in GC rich areas and mature TLS of the TME. B cells evolve towards atypical terminally differentiated, class-switched, and IFN-signaling trajectory endpoints. IFN-rich B cell subsets expressing IgM +/IgD + or class-switched to alternatively-activated isotypes such as IgG4 are localized with T cells and within TLS, and are either retained or induced post-immunotherapy. Future studies applying larger, more focused CyTOF or multiplexed phenotyping panels in expanded cohorts will be important to further validate and refine these B cell populations and their association with therapeutic outcome. Our study reveals evolutional, spatial and interactive B cell behaviors within the TME and links atypical lineage traits and dynamics with immunotherapy. Identification of inflammatory B cell trajectories may be crucial in developing strategies in future to manipulate B cell responses for improved therapeutic outcomes.

## Supplementary Information

Supplementary Material 1.

Supplementary Material 2.

Supplementary Material 3.

Supplementary Material 4.

Supplementary Material 5.

Supplementary Material 6.

Supplementary Material 7.

Supplementary Material 8.

Supplementary Material 9.

Supplementary Material 10.

Supplementary Material 11.

Supplementary Material 12.

Supplementary Material 13.

Supplementary Material 14.

Supplementary Material 15.

Supplementary Material 16.

Supplementary Material 17.

Supplementary Material 18.

Supplementary Material 19.

Supplementary Material 20.

Supplementary Material 21.

Supplementary Material 22.

Supplementary Material 23.

Supplementary Material 24.

Supplementary Material 25.

Supplementary Material 26.

Supplementary Material 27.

## Data Availability

All data generated during this study are included in this published article [and its supplementary information files]. Publicly available datasets analyzed in this study are available in the https://www.ncbi.nlm.nih.gov/pubmed/30595452 and https://www.ncbi.nlm.nih.gov/pubmed/30388456 repositories.

## Abbreviations

BCR: B cell receptor
GC: Germinal center
CSR: Class-switch recombination
SHM: Somatic hypermutation
TME: Tumor microenvironment
TLS: Tertiary lymphoid structure
IFN: Interferon
NK: Natural killer
DC: Dendritic cell
scRNA-seq: Single-cell RNA sequencing
CyTOF: Cytometry by Time-Of-Flight (Mass cytometry)
