# Supplementary material for: Tumor-infiltrating B cells evolve towards interferon-rich trajectories aligned with immunotherapy in melanoma
Source: J Exp Clin Cancer Res. 2026 Aug 7;45:186. doi: 10.1186/s13046-026-03797-1 (PMC13505104; doi:10.1186/s13046-026-03797-1)
Supplement: Supplementary file 1 [file 13046_2026_3797_MOESM5_ESM.pdf]

Supplementary Materials & Methods

1.1 Cell staining, data acquisition and FlowSOM analysis

Thawing of melanoma tumours for mass cytometry analyses

Cryovials of frozen single cell-suspended tumors were thawed in a 37°C water bath. When only a few ice crystals remained, cells were aspirated with a sterile Pasteur pipette and transferred into 10 ml warmed RPMI 1640 + 20% FBS. Cells were washed with 50 ml RPMI 1640 and centrifuged at 300g for 10 minutes. Cells were then incubated 1 hour at 37°C, followed by another RPMI wash as above. PBMC counts were performed on a haemocytometer using trypan blue (1:10 in PBS).

1.2 Mass cytometry cell staining and data acquisition

Thawed cells were placed in filter cap FACS tubes and washed with 3 ml MaxPar Cell Staining Buffer (Standard Biotools) at 800g for 5 minutes. Pellets were resuspended in 80 µl buffer, transferred to FACS tubes, and incubated with 5 µl Fc Blocking Solution (Human TruStain FcX, BioLegend) for 10minutes at room temperature. All antibodies were vortexed and centrifuged at 10,000g for 5 minutes to remove aggregates before use. Surface antibodies were added, samples were gently vortexed and incubated 30 minutes at room temperature. Cells were washed with 1ml MaxPar Cell Staining buffer, and 1uM Rhodium intercalator was used as a viability stain, added to each sample for 15 minutes at RT. Cells were washed as before, and fixed using 1 ml MaxPar Fix I buffer (Standard Biotools) at room temperature for 15 minutes, then washed twice in MaxPar Perm S permeabilisation buffer (Standard Biotools). The intracellular antibody cocktail was added and incubated 30 minutes at room temperature. After a wash in MaxPar buffer, cells were incubated overnight at 4°C in intercalation solution containing DNA intercalator 103 Ir (Standard Biotools) in MaxPar Fix and Perm Buffer. Immediately prior to acquisition, cells were washed in MaxPar Cell Staining Buffer and washed twice in Milli Q water at 800g for 5 minutes. Samples were resuspended in Milli Q water with polystyrene bead standards at 0.5 $\times 10^6$ cells/ml and run on a Helios™ Mass Cytometer.

Mass cytometry and cluster analysis using the FlowSOM clustering algorithm

Raw data were received as .fcs files from the Helios™ Mass Cytometer. Files were normalized and concatenated using Fluidigm CyTOF Software and then uploaded to FlowJo for gating. EQ beads, DNA intercalator, and event length were used to discriminate intact DNA positive singlets from debris

and aggregates. Rhodium live/dead staining identified live intact singlets. B cells were gated as CD45+ CD19+, then refined to exclude doublets using the markers CD45, CD19, CD8a, CD4, CD3, CD16a to define a ‘True’ CD19+ population followed by CD27+. After isolating the memory B cell CD19+CD27+ population in FlowJo, the .fcs files were exported for downstream analysis in R. In R we used a modified script based on the CATALYST, diffCYT, FlowSOM, edgeR, and flowCORE packages. CD27+ B cell phenotypes were defined with an unsupervised FlowSOM clustering algorithm, which uses a self rrganising map to evaluate marker expression in high dimensional space and generate well segregated B cell subsets. Samples pooled for clustering yielded distinct metaclusters, which were annotated according to median scaled expression as previously described (Nowicka et al 2017).
