# Supplementary material for: Tumor-infiltrating B cells evolve towards interferon-rich trajectories aligned with immunotherapy in melanoma
Source: J Exp Clin Cancer Res. 2026 Aug 7;45:186. doi: 10.1186/s13046-026-03797-1 (PMC13505104; doi:10.1186/s13046-026-03797-1)
Supplement: Supplementary file 2 [file 13046_2026_3797_MOESM6_ESM.pdf]

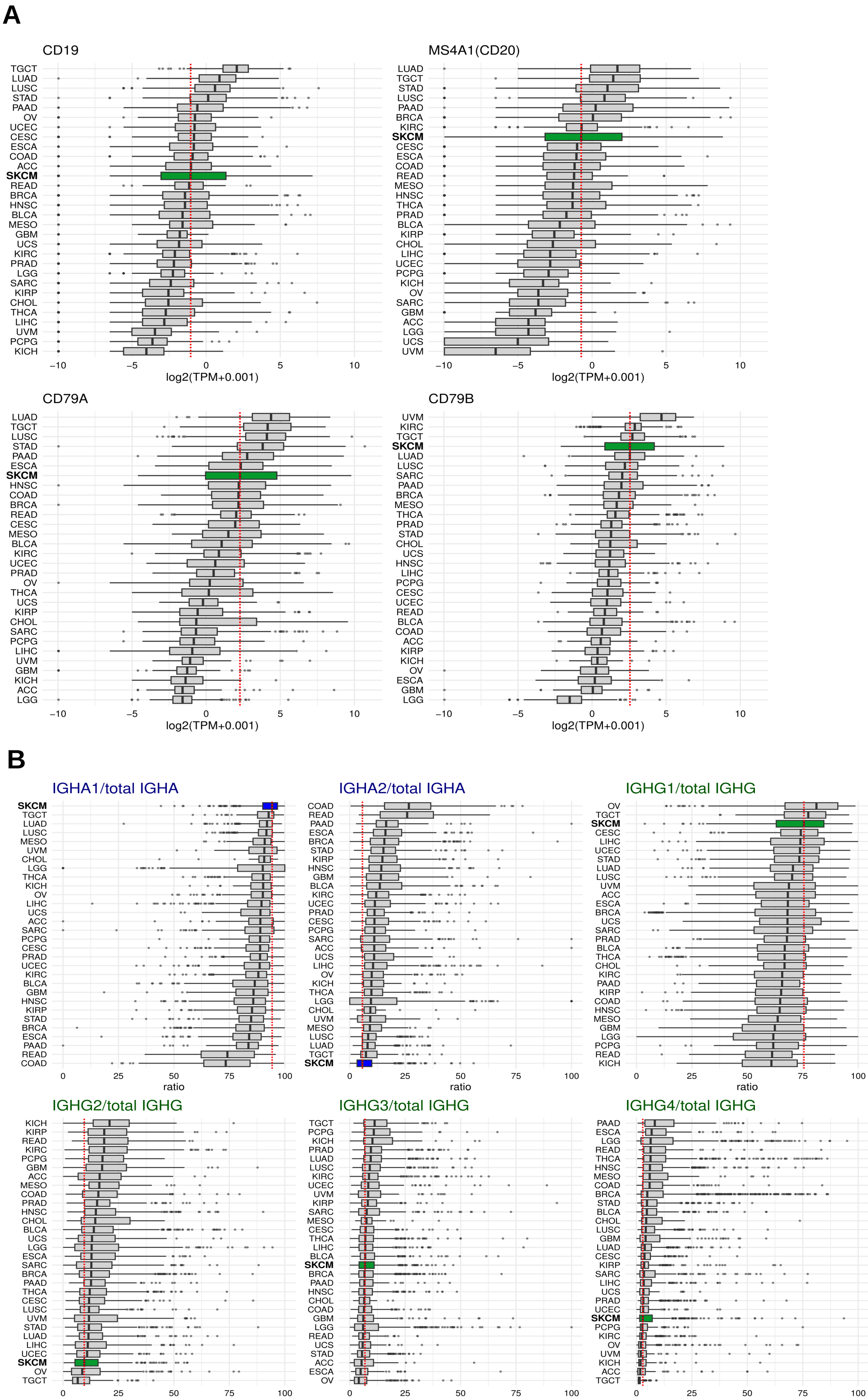

Supplementary Figure 1: B cell marker and immunoglobulin gene expression across bulk RNA-sequencing data from The Cancer Genome Atlas (TCGA). (A) Gene expression of B cell markers and (B) ratios of IGHA subclasses to IGHA and IGHG subclasses to IGHG, demonstrating differences between TCGA samples from different cancers, and highlighting skin cutaneous melanoma (SKCM) (green). Red line represents median for SKCM. Transcripts per million (TPM).

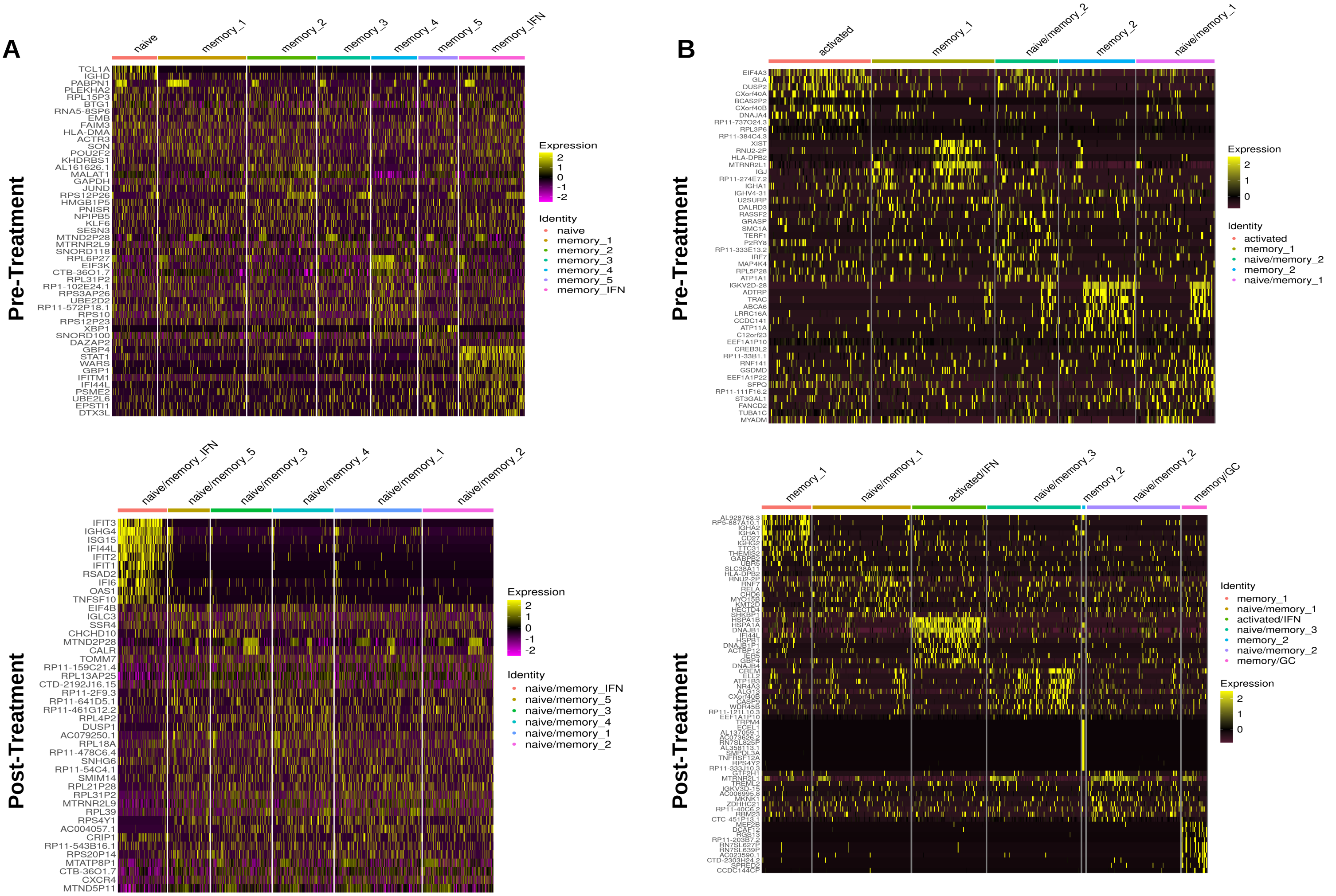

Supplementary Figure 2: Supplementary Figure 2: B cell subtype annotation in single-cell RNA sequencing clusters from GSE123139 and GSE120575 pre- and post-immunotherapy. Heatmaps show differential genes (DEGs) between B cell subsets in pre- and post-treatment melanoma cells from (A) GSE123139 and (B) GSE120575.

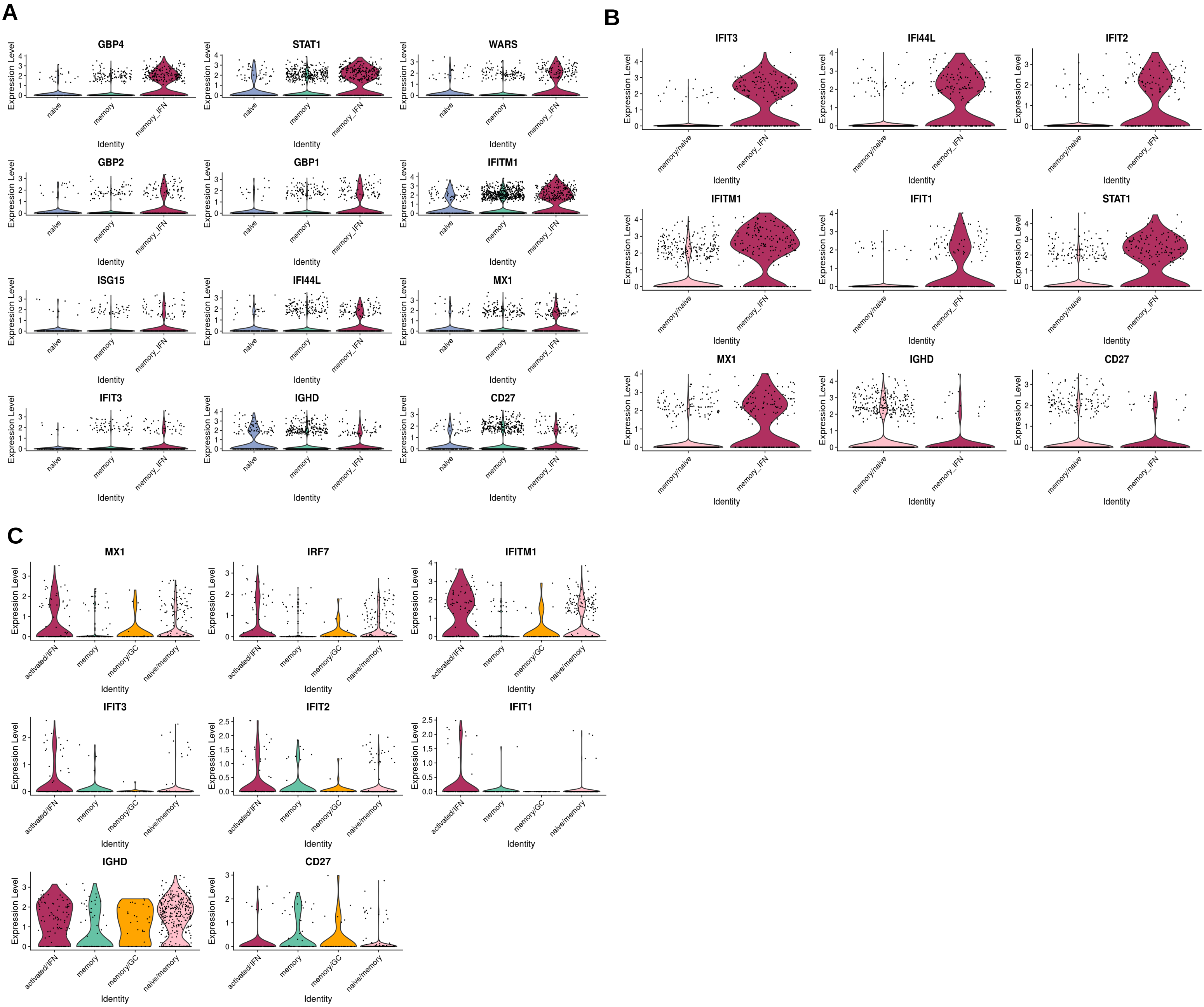

Supplementary Figure 3: Expression of inflammatory genes, IGHD and CD27 in IFN B cell subsets across scRNA sequencing data. Expression of genes in B cell subtype clusters from (A) GSE123139 pre-treatment, (B) GSE123139 post-treatment and (C) GSE120575 post-treatment.

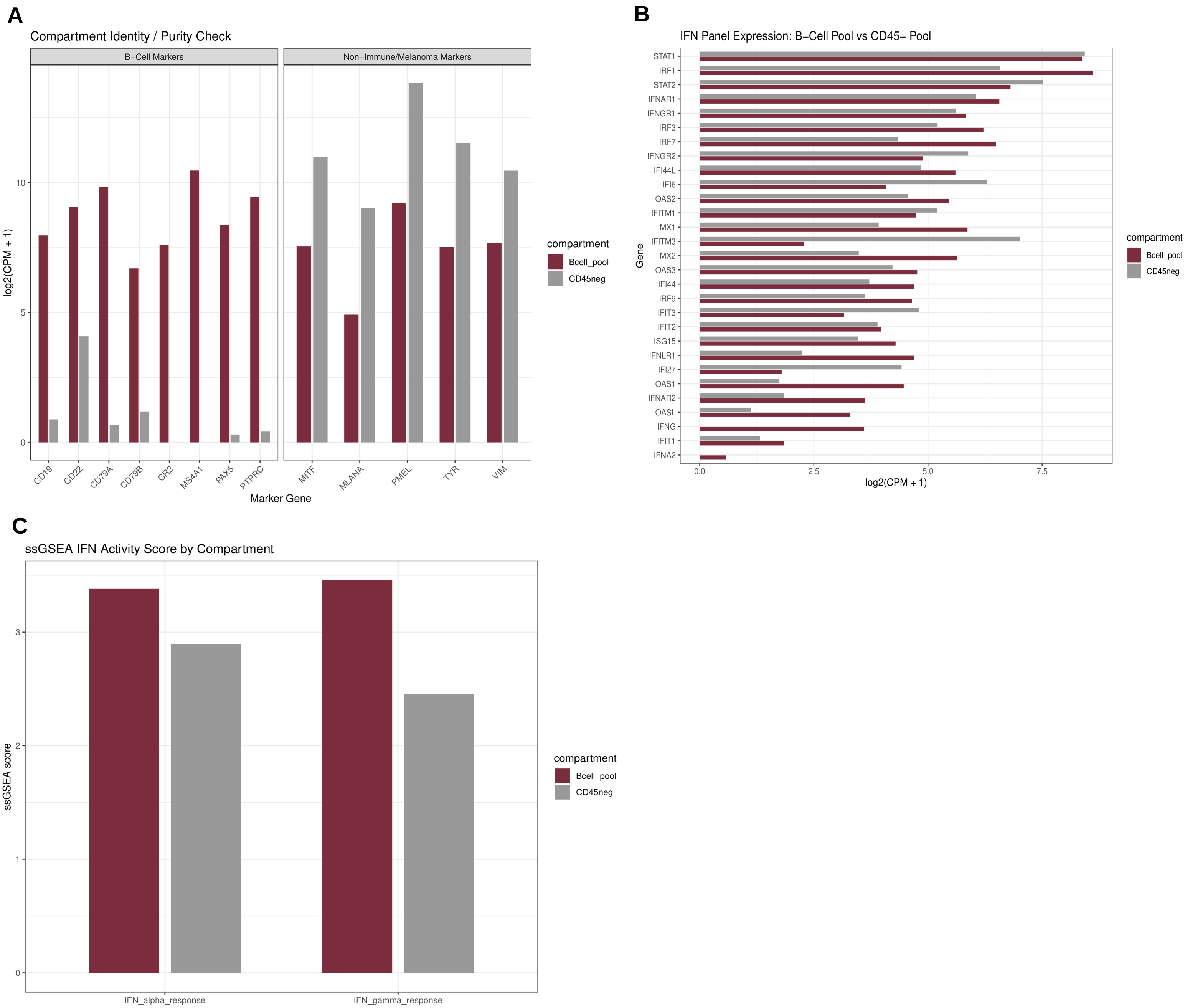

Supplementary Figure 4: Flow cytometry-sorted and pooled B cells from melanoma tumors (n=7) alongside matched CD45- cells from the same specimens. (A) Transcriptomic purity check of sorted B cell and CD45- populations using pan-B cell markers and melanoma-associated markers. (B) Tumor-derived B cells demonstrate proportionally higher expression of multiple IFN-response genes. (C) Gene set variation analysis (GSVA) of IFN-alpha and IFN-beta response signatures in sorted B cells relative to CD45- cells.

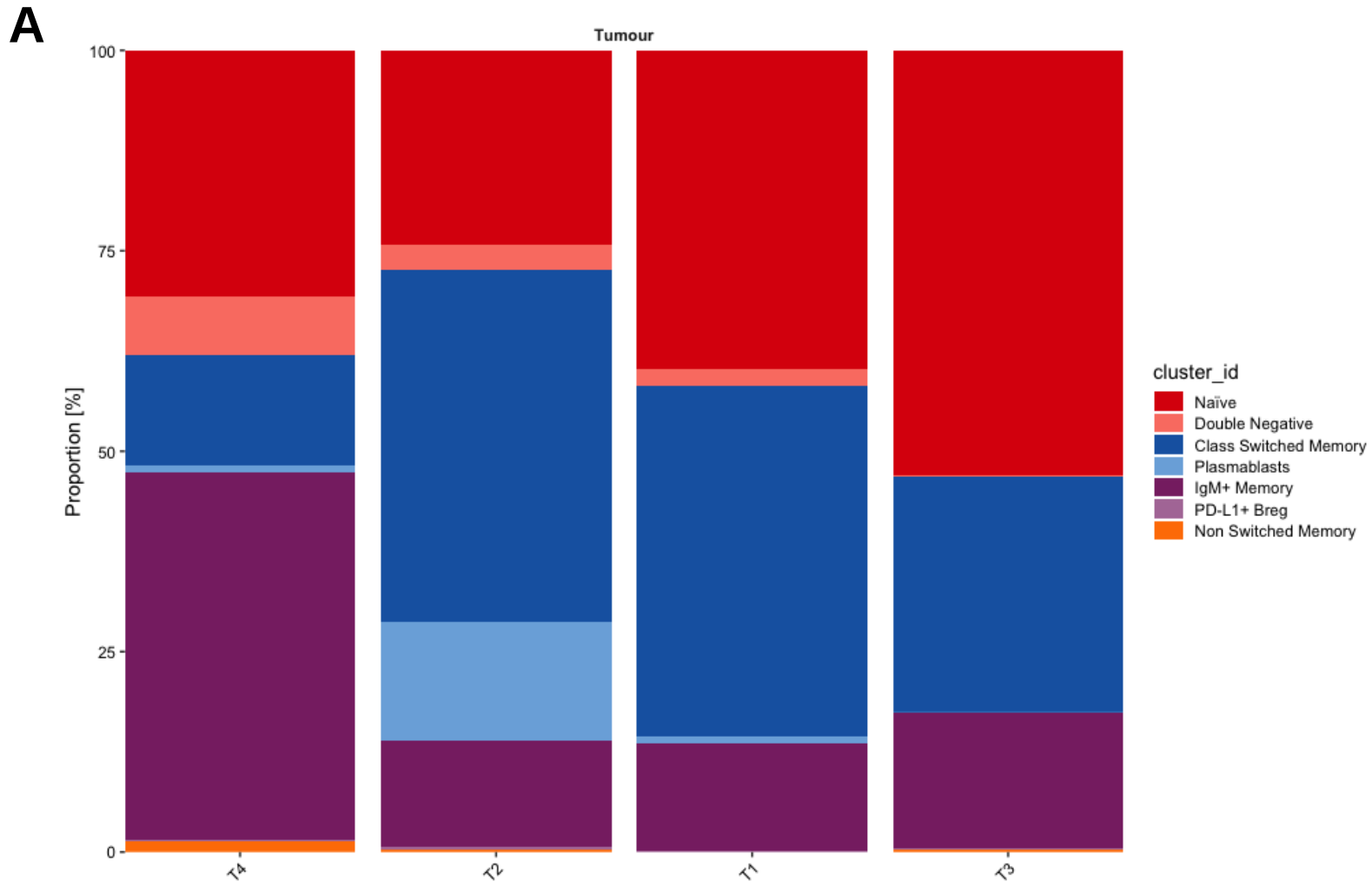

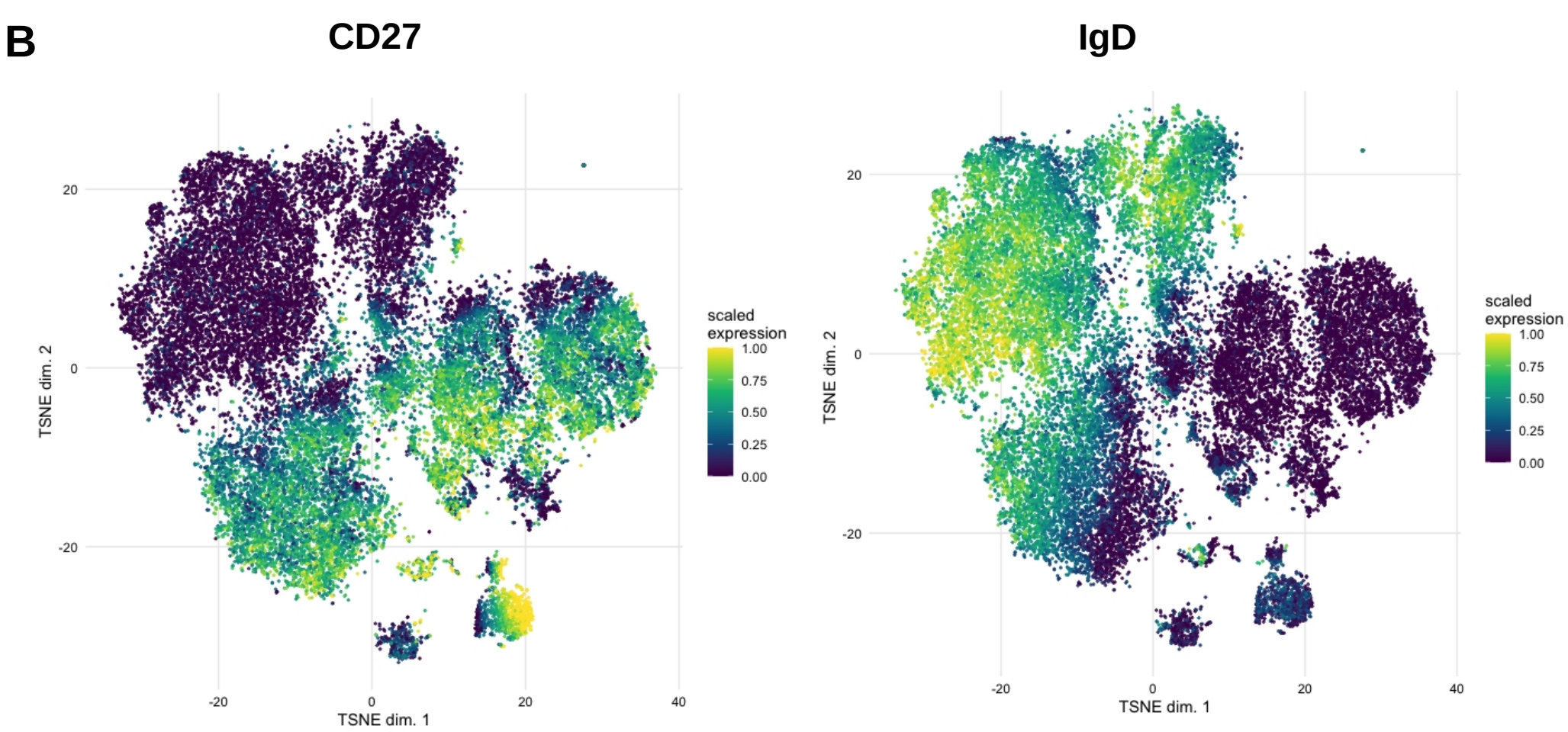

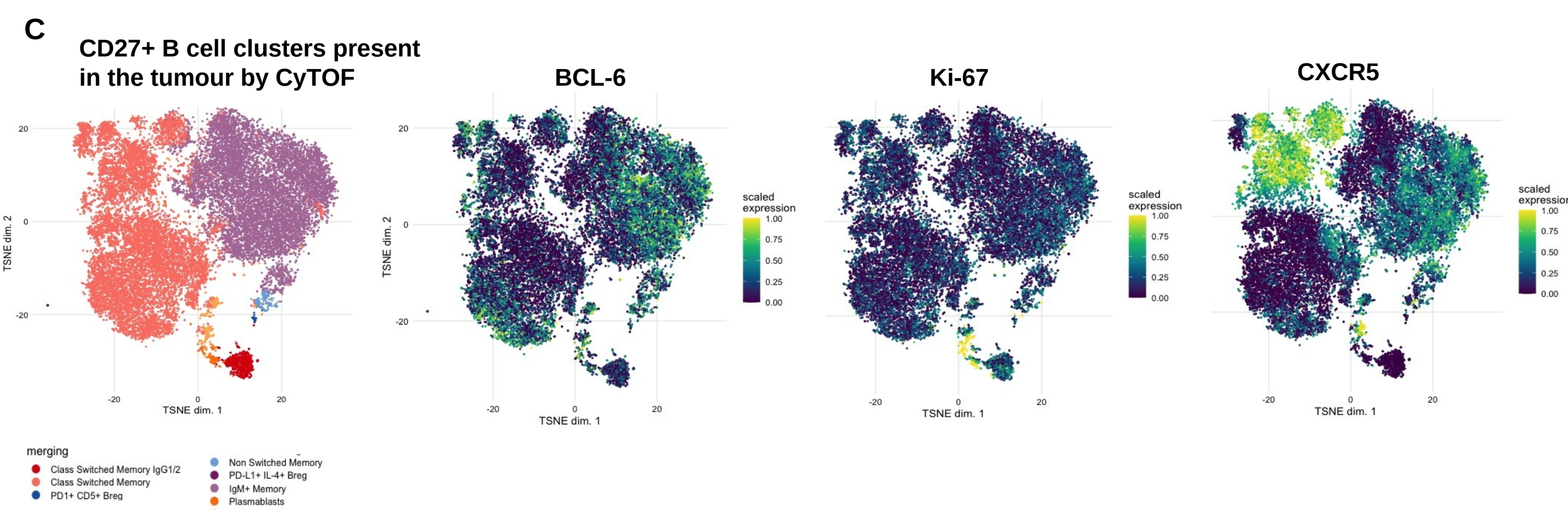

Supplementary Figure 5: Immunophenotyping of CD27+ B cells by CyTOF in melanoma tumors. (A) FlowSOM clustering was used to generate seven B cell subsets from the tumors of four melanoma patients. (B) TSNE showing distribution of CD27+ B cells and IgD+ B cells in the tumor. (C) TSNE showing distribution of CD27+ B cells, and marker expression of key GC (BCL-6, CXCR5) and proliferation (Ki-67) factors.

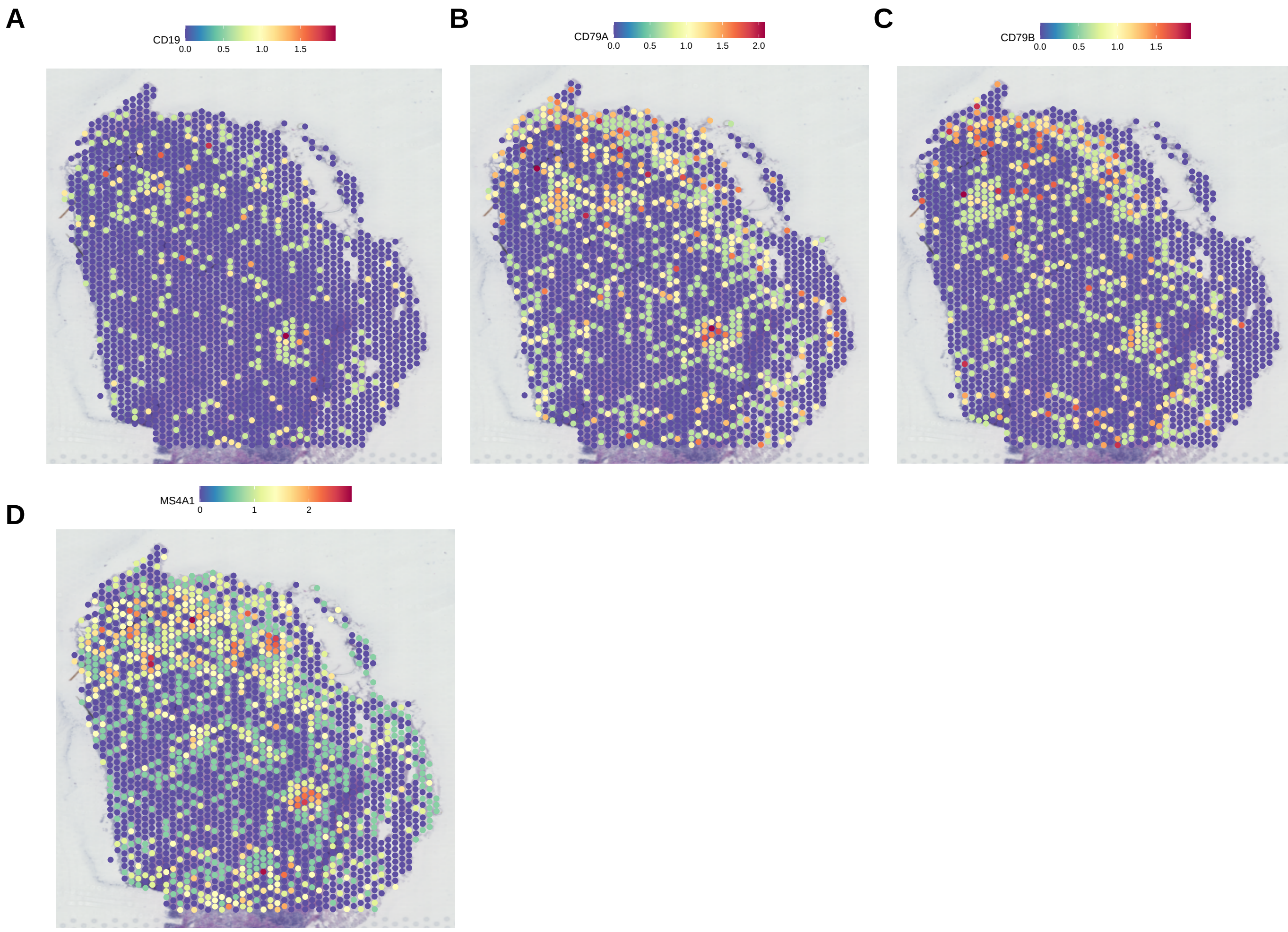

Supplementary Figure 6: Spatial transcriptomic analysis of pan-B cell genes in melanoma tumor. Images include expression of B cell canonical markers including (A) CD19, (B) CD79A, (C) CD79B, (D) MS4A1.

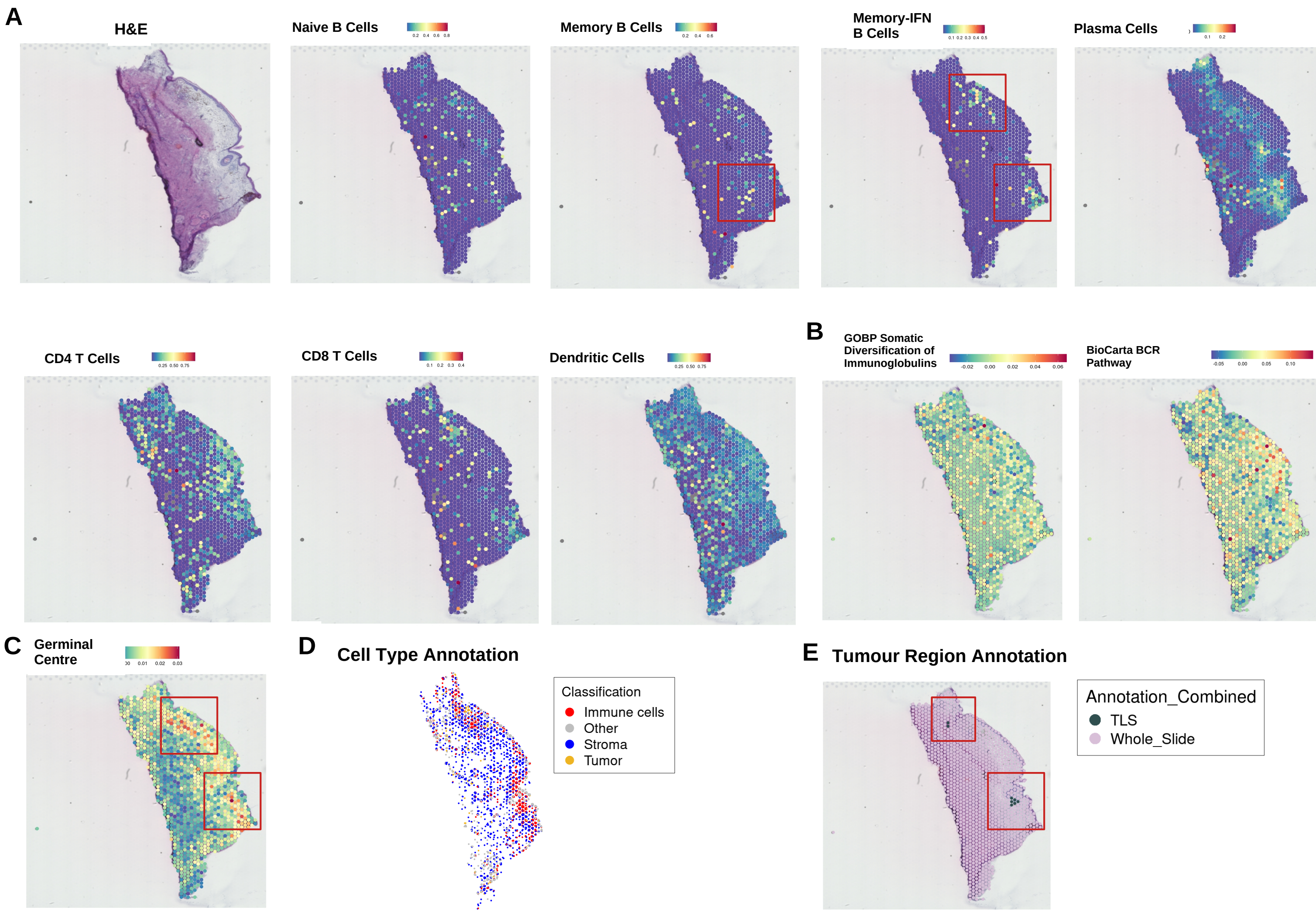

Supplementary Figure 7: Spatial transcriptomic analysis of B cell populations, alongside T cell, germinal center and lymphoid features in melanoma lesions. (A) Representative image M498 of deconvoluted spatial transcriptomic of melanoma sample slices shows infiltration of naive, memory and memory-IFN B cells, CD4+ and CD8+ T cells, plasma cells, and dendritic cells, alongside plasmablast signature enrichment. (B) High enrichment scores were revealed when analysing the expression of the gene sets 'somatic differentiation of immunoglobulins' and 'BCR signalling pathway' from MsigDB. (C) Enrichment of a germinal-centre (GC) signature reveals high GC enrichment score in both B cell localization areas. (D, E) Histological annotation in representative tumor slice shows cell type annotation including immune, tumor and stromal cells (D) and tumor regions including (E) invasive front, tertiary lymphoid structure (TLS) and tumor body.

A **Cell Type (Immune, Stroma and Tumour) Distribution by Tumour Slice**

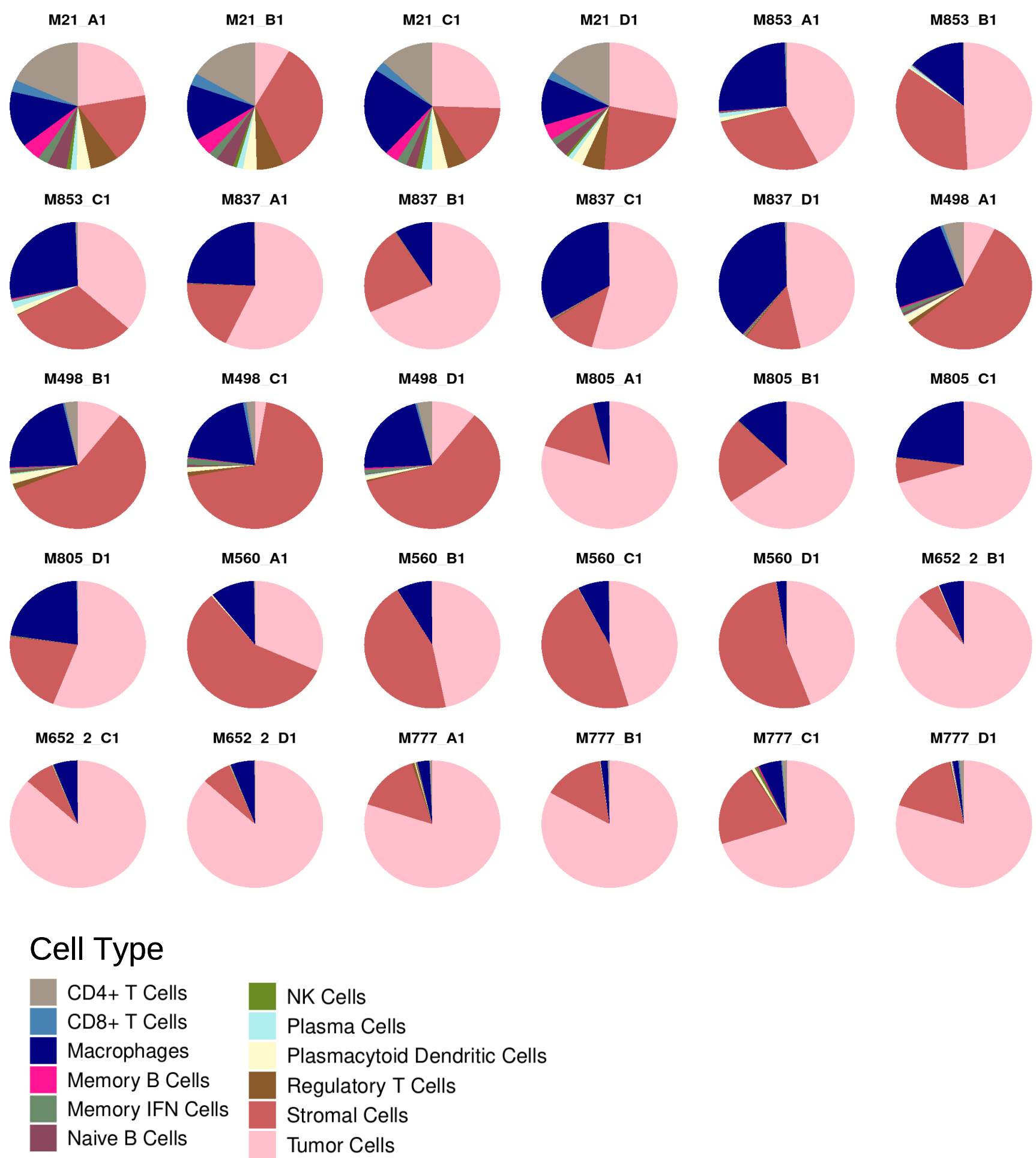

No. of Tumour Infiltrating Immune Cells

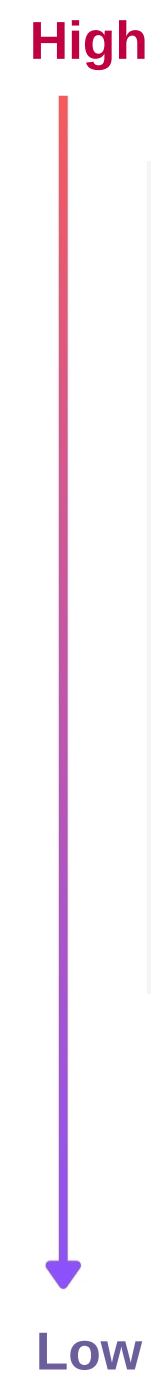

Tumour Sample	Total Cell Count	Total *Immune* Cell Count	Immune % of Total Cells
M21	139, 460	81,949	58.8%
M853	25,162	7,080	28.1%
M837	34,650	9,223	26.6%
M498	27, 970	6,454	23.0%
M805	76,686	12,277	16.0%
M560	53, 372	4,202	7.9%
M652	9,775	717	7.3%
M777	15, 661	1,104	7.0%

Supplementary Figure 8: Quantification of B cell density and B cell subset localisation in tumor lesions by spatial transcriptomic analyses. (A) Pie charts show proportion of cell types in each tumor slice, and No. of Tumor Infiltrating Immune Cells as a proportion of total immune cells.

A

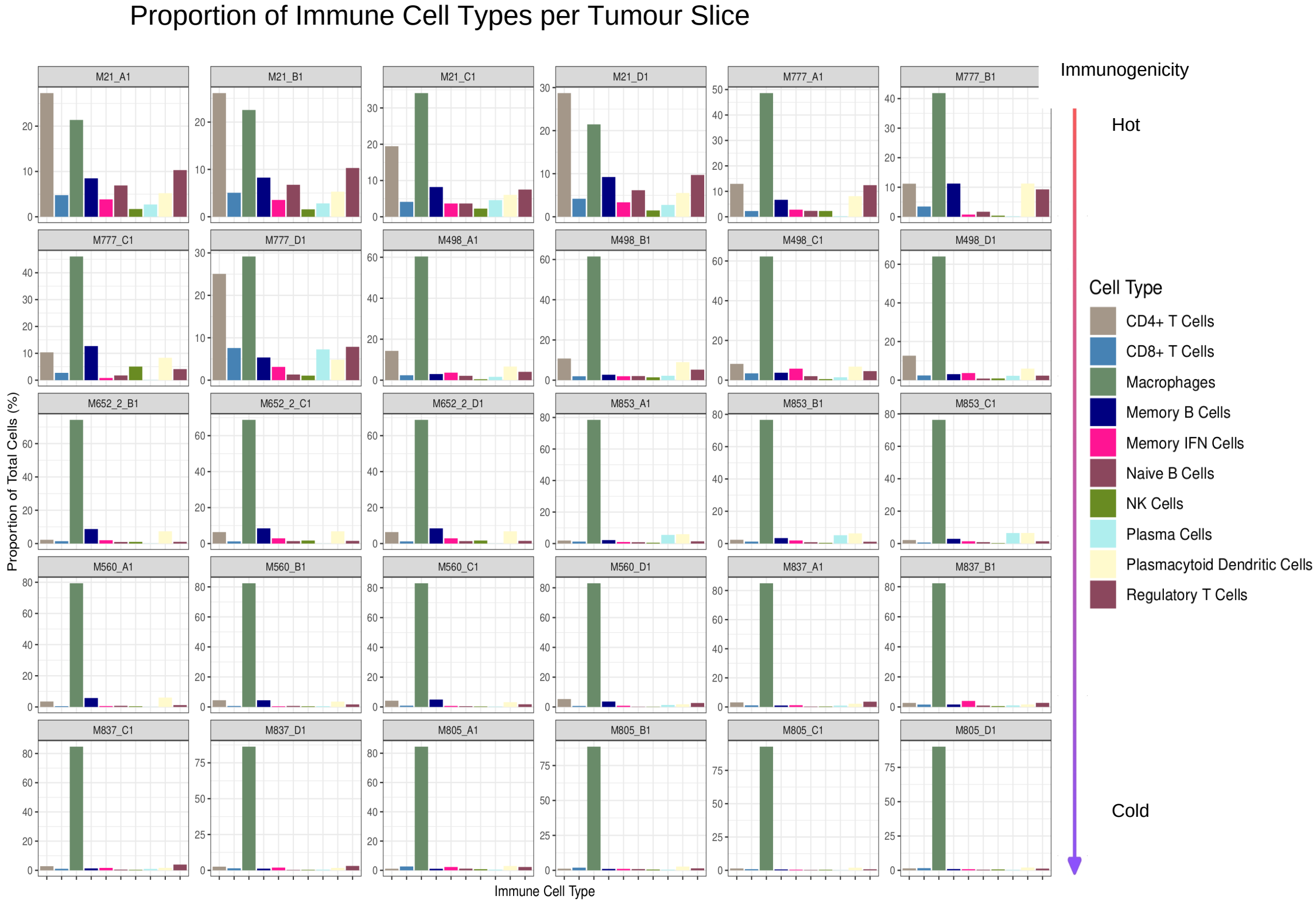

Supplementary Figure 9: Immune cell infiltration in the spatial transcriptomic melanoma tissue slices derived from deconvolution analysis. Immune cell infiltration stratifies samples into immunogenic “hot” and “cold” samples. Infiltration remains unvaried between tumor slices from the same samples.

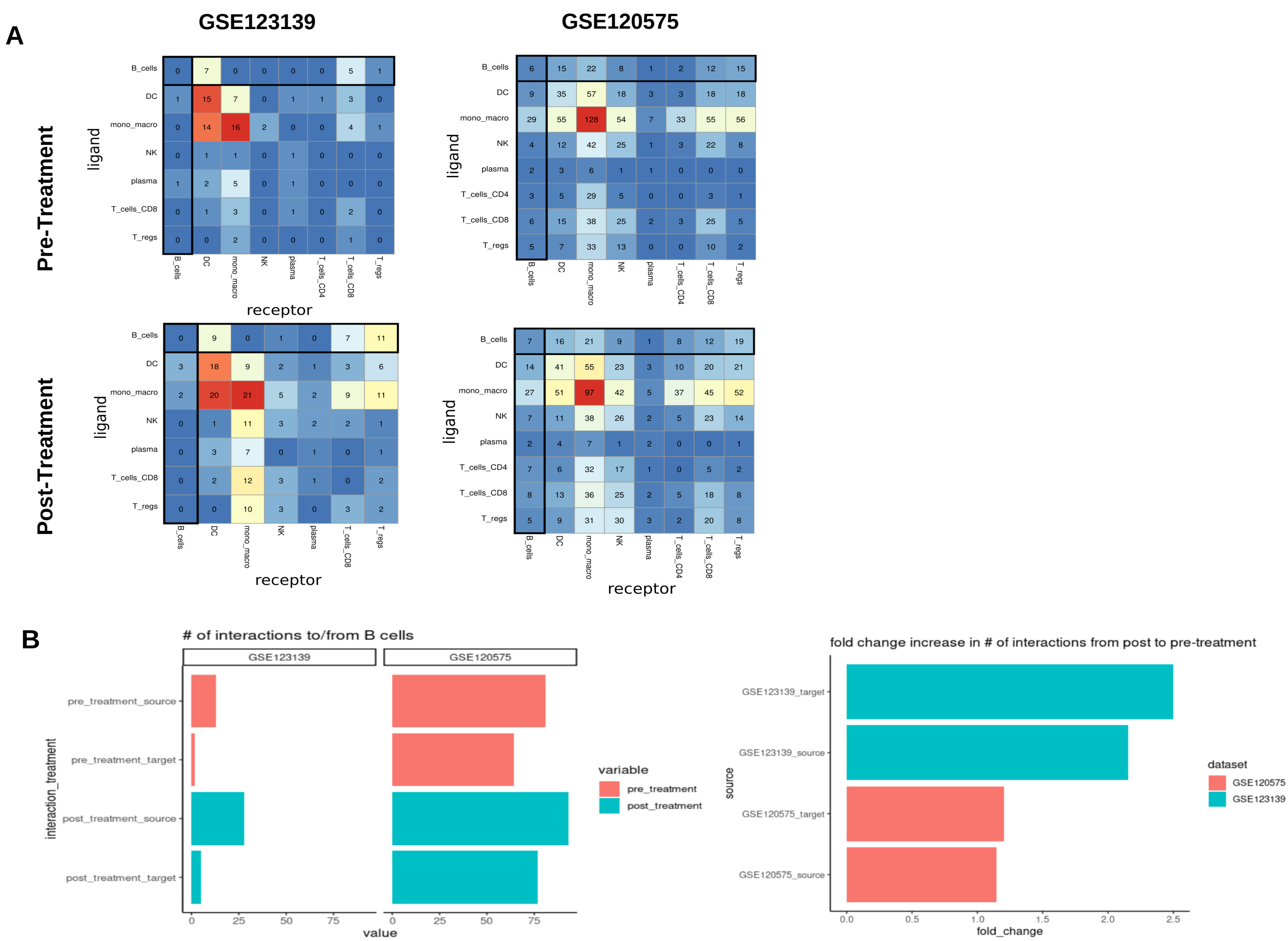

Supplementary Figure 10: Single-cell RNA sequencing analysis reveals B cell populations interactions with other immune cells in pre- and post-treatment samples from melanoma patients. (A) Cell-cell interactions analysis shows the number of significant interactions between immune cell populations providing a ligand or receptor. (B) Left: comparison between the number of significant ligand-receptor interactions where B cells provide a ligand (source) or a receptor (target). Right: the fold change showcasing the increase in the number of significant interaction pairs between B cells and other cell populations after treatment, when compared to the pre-treatment.

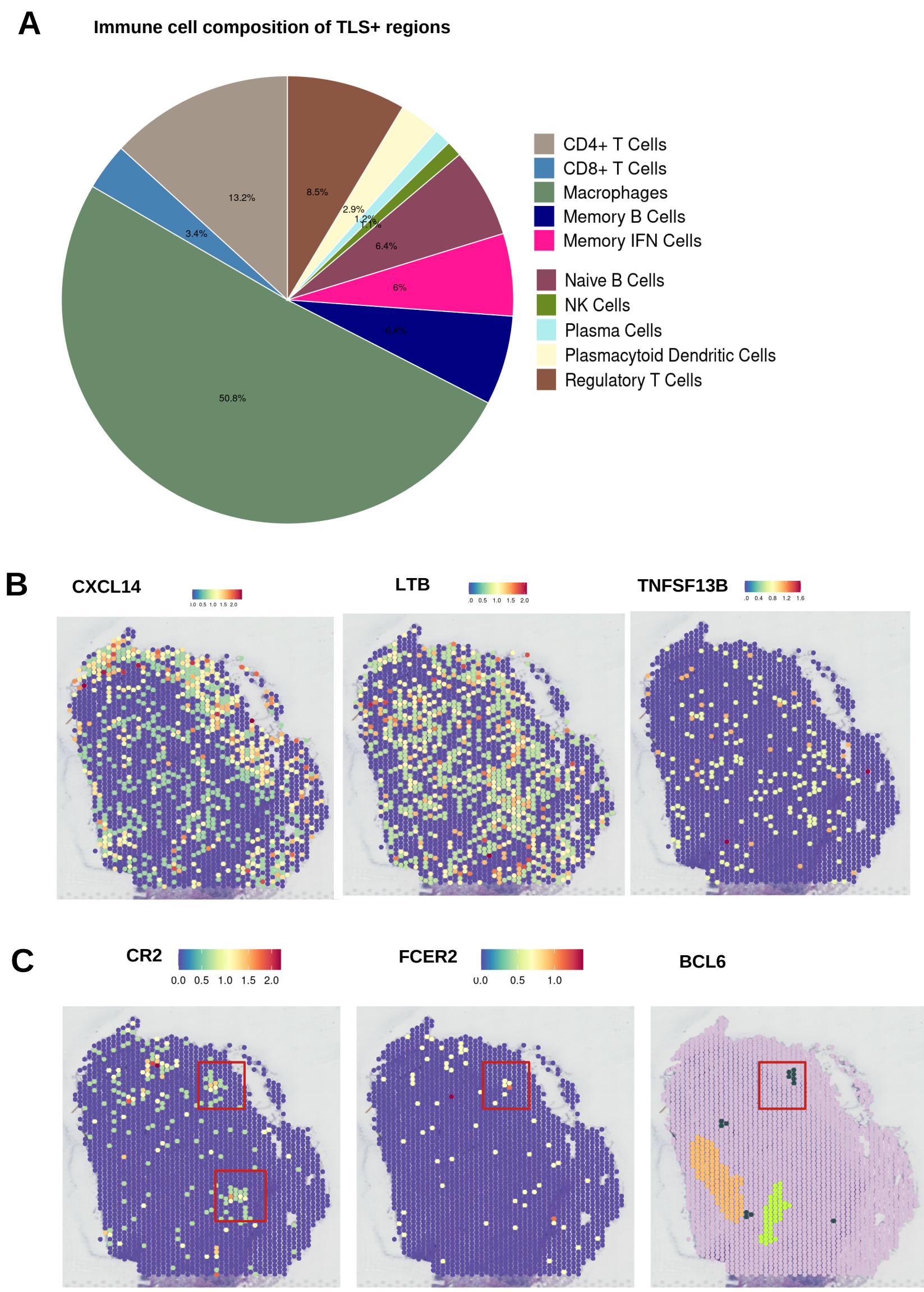

Supplementary Figure 11: Association of B cell subsets in TLS melanoma lesions by spatial transcriptomic analysis. (A) Pie chart shows immune cell composition of TLS+ regions in melanoma lesions. (B) The expression of early TLS markers CXCL14, LTB and TNFSF13B and (C) late TLS markers CR2, FCER2 and BCL6.

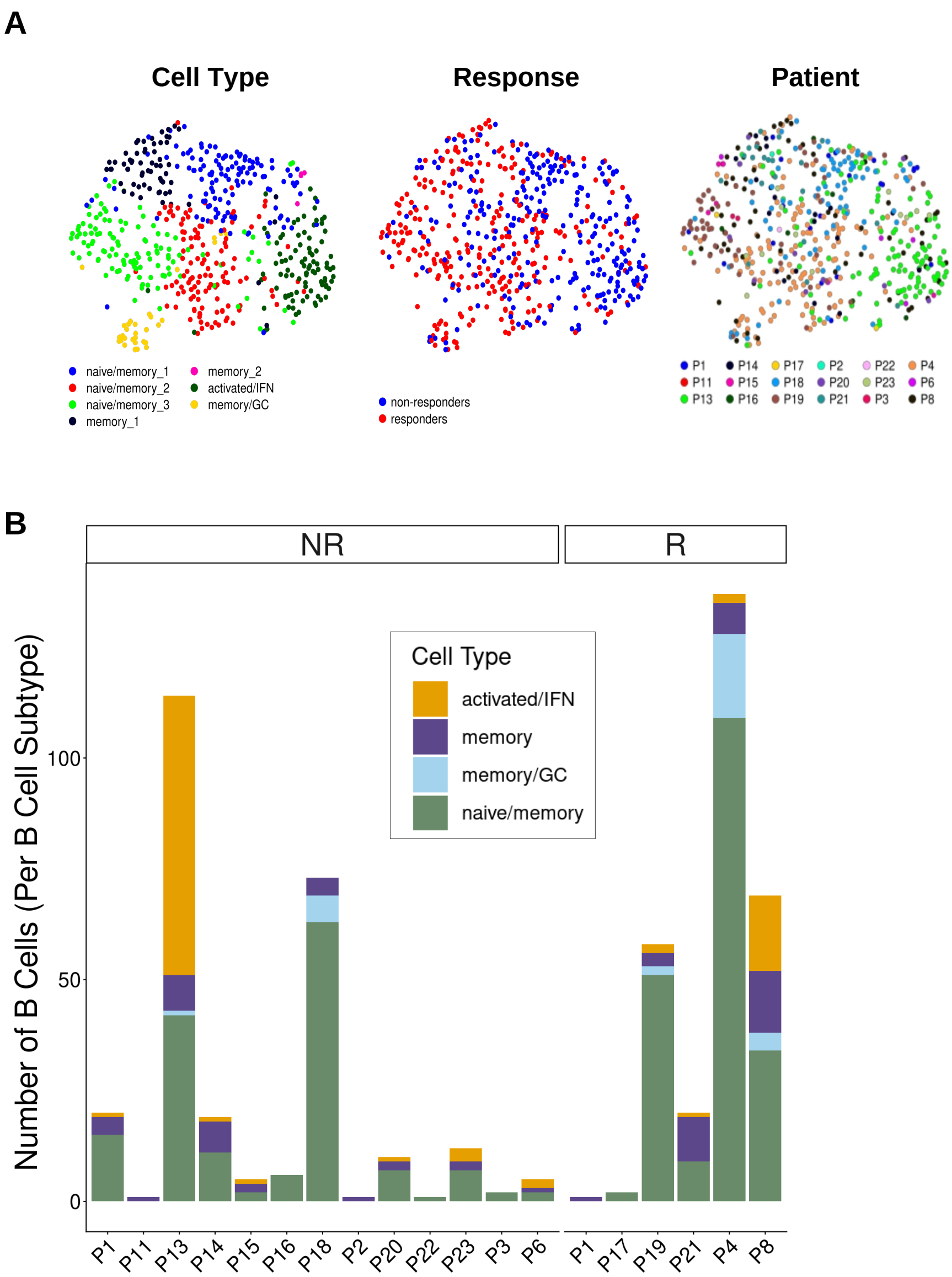

Supplementary Figure 12: B cell subsets distribution across patient ID's from single-cell RNA sequencing data (GSE120575). (A) UMAP projections highlightB cell subtype and response in B cells from post-treated melanoma patient samples. (B) Bar plot shows the number of each B cell subtype in patients from non-responder (NR) and responder (R) patients following immunotherapy treatment.

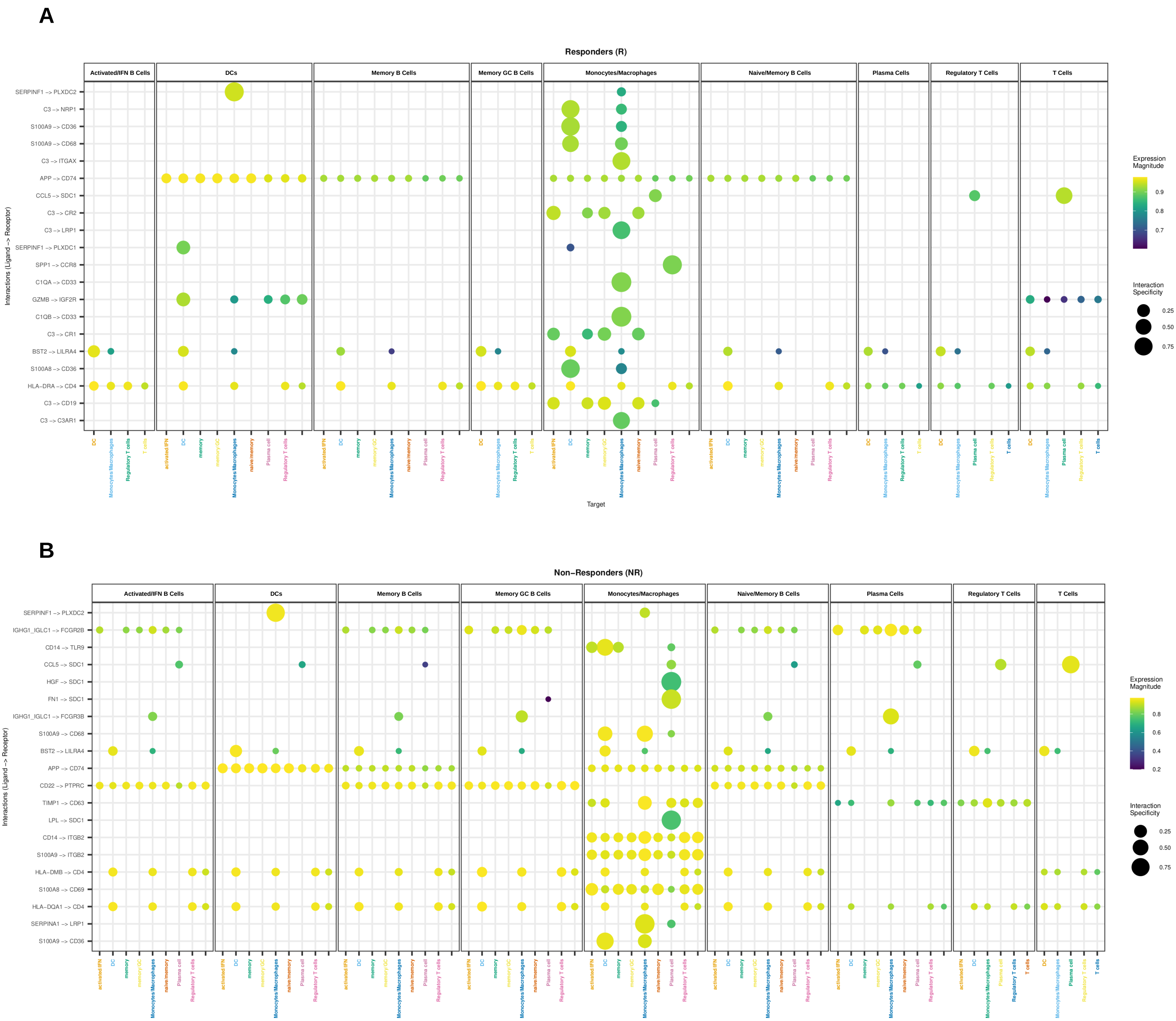

Supplementary Figure 13: Top 20 cell-cell interactions in melanoma tumor samples derived from single-cell RNA sequencing data (GSE120575). Interactions were studied in tumors of (A) Responders (R) and (B) Non-Responders (NR) melanoma patients following immunotherapy.
